# Nano-encapsulated Iron and Folic Acid-Fortified Functional Yogurt Enhance Anemia in Albino Rats

**DOI:** 10.3389/fnut.2021.654624

**Published:** 2021-04-07

**Authors:** Amira M. G. Darwish, Tarek N. Soliman, Hassan A. Elhendy, Wedad M. El-Kholy

**Affiliations:** ^1^Department of Food Technology, Arid Lands Cultivation Research Institute (ALCRI), City of Scientific Research and Technological Applications (SRTA-City), Alexandria, Egypt; ^2^Food Industries and Nutrition Research Division, Department of Dairy, National Research Centre, Cairo, Egypt; ^3^Home Economics Department, Faculty of Agriculture, Alexandria University, Alexandria, Egypt; ^4^Department of Dairy Technology Research, Food Technology Research Institute, Agriculture Research Center, Giza, Egypt

**Keywords:** anti-anemia, natural products, bioavailability, nano-encapsulated Fe and FA, food supplements, stirred functional yogurt

## Abstract

Iron deficiency anemia (IDA) is a major health concern in developing countries, and these see an increased incidence in pregnant women and children in particular. The contribution of dairy products as natural products in drug delivery approaches is inspiring. This study aimed to analyze the application of iron (Fe) and folic acid (FA) bovine serum albumin-nanoparticles (BSA-NPs) as anti-anemic pharmacological agents that fortify stirred functional yogurt (SFY), comparing these with a plain control and SFY fortified with Fe and FA in free forms. The physicochemical, cytotoxicity, microbiological, viscosity, oxidative interactions, microstructural, sensorial analyses, and bioavailability properties of IDA-induced Albino rats were examined. The Transmission Electron Microscope (TEM), Zetasizer, and Scan Electron Microscope (SEM) were applied. Nanocapsule-fortified SFY showed an enhanced apparent viscosity, water-holding capacity, microstructure, least lipid oxidation, and overall sensorial acceptability. Feed that included Fe + FA nanocapsule-fortified SFY (G6) succeeded in restoring hemoglobin (16.53 gdL^−1^), iron (109.25 μgdL^−1^), ferritin (33.25 μgdL^−1^), and total protein (8.6 gdL^−1^) at the end of the 4-week feeding period, with significant competition revealed in calcium and zinc absorbance. Nanocapsule-fortified SFY showed no adverse effects or architectural alterations in the liver, kidney, or spleen, as indicated by biochemical and histological examinations. Bovine serum albumin-nanoparticles (BSA-NPs) of iron (Fe) and folic acid (FA) can be recommended as anti-anemia supplements in different functional food applications.

Iron deficiency anemia (IDA) is one of the top 20 risk factors for the global distribution of disease burden. The WHO is working with the Egyptian government to address major challenges due to the prevalence of IDA, which is found in 40% of children between 2 and 5 years of age and which may increase to 51% in rural areas. A similar prevalence was reported among women of reproductive age and in pregnancy in addition to it being associated with other diseases (1, 2).

Milk is a natural optimized delivery system that provides sufficient bioavailability to sustain life; despite this realization, the use of milk and dairy products in a drug delivery context has not been advanced (3). Yogurt is a dairy product produced by the fermentation of milk with lactic acid bacteria (LAB), and it contains insignificant quantities of iron (Fe) (4) and folic acid (FA) (5). Fortification of yogurt with Fe and FA could decrease nutritional deficiencies, but, unfortunately, it can chemically interact with various yogurt ingredients, causing changes in the physicochemical properties (e.g., syneresis), fat oxidation, resulting in an oxidized and metallic flavor, organoleptic properties (e.g., color and odor), shelf life, viscosity, and microbial properties. Generally, properties of fortified dairy products are influenced by the type, amount, and form of the mineral source component added (6).

The growing awareness of functional foods has increased the need to improve the health benefits of traditional foods, particularly dairy products. This can be achieved by including health-promoting ingredients using nanoencapsulation technology (7). This trend has been driven by the nanostructures' ability to improve the bioavailability and solubility of active ingredients and can be achieved without compromising other food properties (8).

Encapsulation is the method of choice as a technique for confining a substance in a polymeric matrix. It can confer stability of the encapsulated compound more than in its isolated or free forms by protecting from adverse environmental conditions (9), reducing the organoleptic problems, and increasing the bioavailability of ingredients. The mechanism of forming an impermeable membrane as a barrier to oxygen diffusion protects iron from oxidation, masks the flavor and color (10), and enhances bioavailability (11). Encapsulated Fe aided by ascorbic acid was shown to more efficiently maintain the quality of Fe and retain the microbial balance of fortified yogurt (12). Bovine serum albumin (BSA) is a natural biomaterial that is used as a matrix to produce non-toxic, biodegradable, biocompatible, and easily adaptable nanoparticles (NP) (13). BSA is rich in charged amino acids (lysine), which allow the positively and negatively charged molecules to adsorb electrostatically without the participation of any other compounds (14). The desolvation method is a simple and fast technique for the production of protein-based nanoparticles with smaller particle diameters that are considered drug carriers due to their exceptional characteristics: extraordinary adsorption capacity, low toxicity, biodegradability, non-immunogenicity, long-term stability, shelf life, amphiphilicity, and easy scale-up (15, 16).

This study aimed to apply iron (ferrous sulfate) or (ferrous sulfate and FA) bovine serum albumin-nanoparticles (BSA-NPs) as anti-anemic pharmacological agents to fortify stirred functional yogurt (SFY), comparing this with a plain control and SFY fortified with Fe and FA in free forms. Physicochemical, cytotoxicity, microbiological, viscosity, oxidative interactions, microstructural, and sensorial analyses of the fresh products and after 21 days of cold storage were conducted to evaluate the potential changes. Furthermore, the bioavailability and anti-anemia effect of iron-fortified SFY products were studied in IDA-induced male Albino rats.

## Experimental Section

### Chemicals and Materials

Raw cow's milk was obtained from the Faculty of Agriculture farm of Alexandria University, Alexandria Governorate, Egypt. Its composition was 3% fat, 3.1% protein, 12.25% total solids, and 0.172% lactic acid. Skimmed milk powder (SMP) was obtained from Dairy America, Inc., California, USA, and it was composed of 34% protein, 51% lactose, 1.2% fat, 8.2% minerals, and 4% moisture. The other materials used were BSA, FA, L-ascorbic acid, NaCl, and FeSO_4_. 7H_2_O were purchased from Sigma Aldrich, Merck (Sigma Aldrich, Merck St. Louis, MO, USA). All reagents and solvents used were of an analytical grade.

Commercial freeze-dried lactic acid starter cultures for a direct-to-vat set (DVS) included YF-L903, containing *Streptococcus salivarius* subsp. *thermophillus* and *Lactobacillus delbruckii* subsp. *bulgaricus* (1:1), and a probiotic starter culture (ABT-5), which consists of *Streptococcus thermophilus* ST-20Y, *Lactobacillus acidophilus* LA-5, and *Bifidobacterium bifidum* BB-12. Both starter cultures were obtained from Christen Hansen Laboratories, Copenhagen, Denmark, and kept at −18°C. After preliminary incubation, ABT-5 was added to the milk for 18 h at 37 ± 1°C.

### Preparation and Purification of BSA-NPs

Bovine serum albumin nanoparticles (BSA-NPs) were prepared using the previously reported desolvation technique (15). This method is a thermodynamically driven self-assembly process used for polymeric materials. Both hydrophilic and hydrophobic drugs can be encapsulated into NPs using this technique (17, 18). BSA powder (200 mg) was dissolved in 2 mL deionized water. We ensured pH was 7.2 using 0.01 M NaOH, and the solution was left to stir at 500 rpm at room temperature (25°C) for 10 min to equilibrate. Subsequently, by continuous dropwise addition of 8.0 mL ethanol by a syringe pump at the rate of 1.0 mL/min as a desolvating agent, an opalescent suspension was achieved, which indicates the formation of the NPs (step i). Ethanol changes the tertiary structure of the protein, and, during the addition of ethanol to the solution, the albumin is phase-separated due to its diminished water solubility (19). Since the formed NPs were not sufficiently stabilized and could consequently redissolve again after dispersion with water, cross-linking was implemented, which is a major step in the desolvation method. In this step, 37.5 mL of 50% aqueous solution of glutaraldehyde (0.2 mL 50% Gta per mg of BSA) (20) was added gradually for the stabilization and cross-linking of the amino moieties in lysine residues and the guanidine side chains in arginine of BSA *via* a condensation reaction with the aldehyde group (step ii). The mixture was maintained under stirring conditions for 12 h.

In order to eliminate the non-desolvated albumin, the excess cross-linking agent, and organic solvent, the resulting NPs were purified by three successive centrifugations (16,000 rpm, 20 min). The first centrifugation supernatant was used for the determination of non-desolvated albumin. After that, between each centrifugation, the supernatant solution was thrown away, and the pellets were washed with the original volume of deionized water (step iii). Then, the redispersion step in deionized water (20 mL) was performed in an ultrasonic bath for 30 min (step iv). The product was dried in a freeze dryer with a cycle of 24 h at a shelf temperature of 55°C and then incubated at 4°C in the dark (step v). Average particle sizes were measured by transmission electron microscopy (TEM), and the samples were dispersed in distilled water for dynamic light scattering (DLS) analysis (21).

## Characterization of BSA-NPs

### Transmission Electron Microscopy

Samples of nano-encapsulated Fe and FA were prepared for transmission electron microscopy (TEM). The samples were diluted (1:100 v/v) with deionized water. A drop of the diluted suspension was placed on the format-coated electron microscopy grid, left for 1 min, and then a drop of phosphotungstic acid solution (2% at pH 7.2) was added. The grid was air-dried and examined by TEM using a JEOL JEM-1400 plus TEM with an accelerating voltage of 100 kV at a magnification of 200,000 x (21).

### Particle Size and Zeta Potential

The nanocapsules' particle size was measured using a dynamic light scattering instrument (DLS) (Mastersizer 2000, Malvern Instruments, Malvern, UK). The particle size of each sample was represented as the surface-weighted mean diameter (d32), which was calculated from the full particle size distribution. The droplet charge (zeta potential) of the nanocapsules was measured using particle microelectrophoresis (Zetasizer Nano ZS-90, Malvern Instruments, Worcestershire, UK) (21).

### Analysis of Fe and FA Loading to BSA-NPs and Encapsulation Efficiency

The BSA-NPs were loaded with Fe and FA, and the loading efficiency was evaluated. The loading process was done by the preparation of nanoparticle solutions by dissolving 25 mg in 1 mL deionized water. Then, the volumes of 1, 2, and 4 mg Fe or 0.025, 0.05, and 0.1 mg FA were added to the nanoparticle solutions; the final volume was adjusted with deionized water to 2 mL and was magnetically stirred for 12 h (600 rpm) at room temperature. After this, there is an adsorption equilibrium between the Fe or FA and the surface of the NPs. These suspensions were transmitted to polypropylene centrifuge tubes and centrifuged at 12,000 rpm for 20 min. The supernatants were separated and were measured using atomic absorption for Fe or HPLC for FA to determine the loading efficiency (21).

### Cytotoxicity Assessment by Hemolytic Activity Assay

Ascorbic acid has been reported to increase Fe absorption by several folds due to its chelating action and reducing power that facilitates ferric (Fe^3+^) conversion to ferrous ions (22). Ascorbic acid was added to the free and nano-encapsulated fortification forms at a concentration of 225 mgL^−1^. Before application, the cytotoxicity of the fortification materials, free ferrous sulfate + ascorbic acid (45 mgL^−1^), free ferrous sulfate (45 mgL^−1^) + free FA (1 mgL^−1^) + ascorbic acid, Fe@-BSA-NPs + ascorbic acid (45 mgL^−1^), and Fe + FA@-BSA-NPs + ascorbic acid (45 mgL^−1^) were assessed by hemolytic activity assay in 2 mL microtubes according to Farias (23) with some modifications. Two-fold serial dilution of each sample was prepared with 0.9% NaCl ranging from 1,000 to 1.9 μg·mL^−1^ and reserved. Then, 100 μL of a 1% red blood cell (A, B, and O human blood types using rabbit blood) suspension were added to a new microtube containing 900 μL of each sample dilution, incubated at 37°C/1 h, and then centrifuged at 3,000 × g/5 min. The supernatant (200μL) was placed in a 96-well plate and led to a microplate reader to measure the absorbance at 540 nm. The cell suspensions of each human blood type (100 μL) were mixed with distilled water or 0.9% NaCl (900 μL) to obtain the absorbance of 100 and 0% of cell lysis, respectively. The percentage of hemolysis was calculated according to the following Equation (1):

(1) $$\begin{array}{r} \%\;hemolysis\;=\;\frac{Abs\;test}{Abs\;pc\;}\times \;100 \end{array}$$

where Abs test = Abs540 of the 1% cell suspension treated with sample test, and Abs *pc* = Abs540 of the 1% cell suspension treated with distilled water.

To calculate the relationship between percentages of hemolysis and sample concentration, the hemolytic activity was expressed as the lowest sample concentration (μg·mL^−1^) capable of causing hemolysis ≥20%. All determinations were run in triplicates.

## Characteristics of Manufactured SFY

### SFY Preparation

Cow's milk was standardized to 14% total solids by adding 3% SMP and then homogenized at 2,500 psi with an Ultra Turrax blender (IKA, Merck, Germany) at 14,000 rpm until all ingredients were dissolved in the milk. The milk was heated to 90°C for 5 min then cooled to 42 ± 1°C. An ABT-5 probiotic starter culture was enriched in SMP (0.05/Kg w/w) for 18 h at 37 ± 1°C and then used to inoculate the warmed milk with the yogurt starter culture (0.03/Kg w/w) at 42 ± 1°C (24). The milk was then was divided into five equal batches for the five treatments and incubated at 42 ± 1°C until a firm curd was obtained. The treatments were as follows: C, Control plain SFY; T_1_, SFY fortified with free Fe + ascorbic acid; T_2_, SFY fortified with free Fe + FA + ascorbic acid; T_3_, SFY fortified with Fe@-BSA-NPs + ascorbic acid; and T_4_, SFY fortified with Fe +FA @-BSA-NPs + ascorbic acid. The curd was then refrigerated at 4°C overnight before being stirred, packaged, and immediately transferred to a refrigerator at 4 ± 1°C for 21 days (25). The SFY products were analyzed for their physicochemical, microbiological, viscosity, oxidative interactions, microstructural, and sensory properties on the first day after manufacture and during the storage period.

### Physiochemical Analysis of SFY

The pH was measured using a digital pH meter (Persica pH 900, Switzerland). According to AOAC 947.05, the titratable acidity (TA) as the % lactic acid was estimated (26).

The water-holding capacity (WHC) was determined using the method reported by (27).

The SFY apparent viscosity was measured in fresh products and at 21 days of cold storage, using a Bohlin coaxial cylinder viscometer (Bohlin Instrument Inc., Sweden) attached to a workstation loaded with V88 viscometer programming software. The viscometer probe, system C30, was placed in the yogurt sample cup, and measurements of viscosity were carried out at 20 ± 2°C in the up mode at shear rates ranging from 19 to 126 s^−1^ (28).

The SFY color analyses were conducted for the fresh products and at 21 days of cold storage using a Hunter colorimeter (Hunter Ultra Scan VIS). Values were expressed by Hunter L, a, and b values where L^*^ was the value of the lightness (0–100 representing dark to light), a^*^ was the value of the degree of red and green color, where a higher positive value was indicated by more red, and b^*^ was the value of the degree of the yellow and blue colors, where higher values were indicated by more yellow (29).

### Phenolic, Flavonoid Content, and Antioxidant Potentials

The total phenolic content (TPC) expressed as a gallic acid equivalent in the μg/g sample was determined by the Folin–Ciocalteu method (30). Total flavonoid content (TPC) was assessed *via* the colorimetric method as described by Sakanaka et al. (31). The results were expressed as μg of catechol equivalent per g of sample. The 2,2-diphenyl-1-picrylhydrazyl (DPPH) assay was performed as described by Brand-Williams et al. (32). Antioxidant activity was expressed as IC_50_ (mgmL^−1^) where the inhibition percent of the DPPH radical was 50%.

### Evaluation of Lipid Oxidation Thiobarbituric Acid Reactive Substances and Peroxide Values

The lipid peroxidation changes were assessed in SFY samples throughout a cold storage period of 21 days. The SFY samples were analyzed for thiobarbituric acid reactive substances according to Radha Krishnan et al. (33). A total of 5 g of the sample were homogenized with 15 mL of deionized distilled water; then, 1 mL of the sample homogenate was transferred into a test tube and 50 μL of butylated hydroxytoluene (7.2%) in methanol and 2 mL of thiobarbituric acid (TBA)–trichloroacetic acid (TCA) (15 mM TBA−15% TCA). The mixture was vortexed and then incubated in a water bath 100°C/15 min to develop color. Samples were subjected to cooling for 10 min, vortexed, and centrifuged at 5,000 rpm/15 min. The absorbance of the resulting supernatant solution was determined at 531 nm against a blank containing 1 ml of deionized water and 2 mL of TBA–TCA solution. The amount of TBARS was expressed as milligrams of malonaldehyde per kilogram of the sample.

The peroxide values (PVs) of the stirred yogurt samples were determined according to official method AOCS (34) (method Cd8-53) (34) by titration, and standard sodium thiosulfate (0.01 N) was calculated as mL equivalent peroxides per kilogram sample (meqO_2_Kg^−1^ sample).

### Microbiological Analysis of SFY

Appropriate serial dilutions of SFY samples were prepared for microbial enumeration by using 2% sodium citrate. For the enumeration of *S. thermophilus*, counts were performed on M17 agar (Biolife, Italy) and incubated at 37°C for 48 h under aerobic conditions. The *L. bulgaricus* was enumeration on MRS agar (Biolife, Italy) and incubated aerobically at 37°C for 72 h (35). The *L. acidophilus* counts were determined using MRS-bile (MRS agar prepared with 1.5 gL^−1^ bile agar.). Selective enumeration of *B. bifidum* was performed on MRS-cysteine agar prepared with 0.05 gL^−1^ cysteine (36). The *B. bifidum* and *L. acidophilus* culture plates were incubated at 37°C for 72 h under anaerobic conditions using a gas pack (Oxide, UK). The enumeration of yeasts and molds was performed as recommended by Okoye and Animalu (37) using potato dextrose agar (Difico, Italy) acidified with 10% tartaric acid and incubated at 25°C for 5 d. Violet red bile lactose agar (Oxide, UK) was used for the coliform count according to Marth (38). The plates were incubated at 37°C for 24 h. The colony-forming units were measured as Log_10_ CFUg^−1^.

### Microstructural Characterization

The microstructures of the SFY samples were examined by (SEM) (Jeol JSM-6300 F, Japan at 2.5–5.0 kV) according to Munir (39). Cubes (3 ± 0.5 mm^3^) were cut from different areas of the yogurt cup and fixed in 3% glutaraldehyde in 0.05 M phosphate buffer pH 7 for 2 h at 48°C. The fixed cubes were rinsed with 0.05 M phosphate buffer followed by the primary fixing solutions, and finally soaked a secondary fixation solution (1.5% osmium tetroxide in phosphate buffer pH 7.0) for 1.5 hrs. The fixed cubes were rinsed with 0.05 M phosphate buffer. The fixed cubes were dehydrated by successive soaking in 30, 50, 70, and 95% ethanol each for 20 min and finally by two rinses in absolute ethanol (100%) at 48°C and 58°C, respectively. Cubes were immediately dried in the critical point drier (Samdri PVT-3B, Tousimis, Rockville, MD) for 5 h. using CO_2_. The dried cube was fractured and mounted on sputter. The analysis was carried out after 7 days of manufacture of yogurt samples.

### Sensory Evaluation

The sensory evaluation of SFY samples was assessed by 20 consumer-oriented panelists (11 men and 9 women aged between 27 and 51 years). Under the supervision and approval of the Institutional Committee, the assessment was conducted at Food Technology Department, Arid Lands Cultivation Research Institute, SRTA-City, Alexandria, in fresh products and after 21 days of cold storage (40, 41). The criteria for selection depended on their experience and background related to yogurt products. The samples, which were stored at (4°C), were allowed to rest at room temperature (25°C), 10 min before evaluation. The samples were evaluated using a 9-point Hedonic scale (42). This scale consisted of the test parameters of taste, odor, body and texture, appearance and color, and overall acceptability, and it was accompanied by a scale of nine categories: 1 = dislike extremely; 2 = dislike much; 3 = dislike moderately; 4 = dislike slightly, 5 = neither dislike nor like, 6 = like slightly; 7 = like moderately; 8 = like much; 9 = like extremely.

## Biological Experiment

### Animals and Experimental Design

A total of 36 male albino rats (150 ± 10 g) were bred and maintained in Experimental Animals House, Home Economics Department, Faculty of Agriculture, Alexandria University, Egypt, after approval of Alexandria University Ethical Committee (AlEXU-IACUC), a member of International Council for Laboratory Animal Science (ICLAS) (Permission number: AU08200415362). All experiments were performed in accordance with Alexandria University Ethical Committee guidelines and regulations. Animals were housed in stainless steel wire-mesh cages in a room maintained at 22 ± 1°C. Rats were acclimatized to being fed laboratory chow (with chemical composition as follows: fat 2.8%, protein 18.5%, and fiber 11.2%) and water *ad libitum* for 1 week to stabilize their metabolic condition. After the adaptation week, the rats were randomly divided into two groups of six animals each. Group I (G1) was the negative control group, and Group II was induced for IDA by feeding them a diet containing 20 g/kg of body weight of tannic acid for 2 weeks. Group II was then randomly divided into five sub-groups—G2, Positive control group; G3, fed SFY fortified with 50 mgKg^−1^ free ferrous sulfate + 125 mgKg^−1^ ascorbic acid; G4, fed SFY fortified with 50 mgKg^−1^ free ferrous sulfate + 0.5 mgKg^−1^ free FA + 125 mgKg^−1^ ascorbic acid; G5, fed SFY fortified with 50 mgKg^−1^ Fe@- BSA-NPs + 125 mgKg^−1^ ascorbic acid; and G6, fed SFY fortified with 50 mgKg^−1^ Fe + FA@ BSA-NPs + 125 mgKg^−1^ ascorbic acid—for another 4 weeks as described by Afsana et al. (43).

### Samples Collection and Preparation

Body weights were monitored once/week. Blood analyses were obtained from rat eyes. At the end of 42 days period and under the approval of the ethical committee, final weights were recorded, and rats were sacrificed after overnight fasting under light diethyl ether anesthesia. Blood samples were collected from the abdominal aorta in plain tubes, centrifuged at 4,000 rpm/20 min at 4°C for serum separation, and stored at −20°C until analyzed. Immediately after necropsy, the rats' organs (liver, kidney, spleen, heart, and lungs) were dissected carefully and weighed, and the weights were recorded and then fixed in 10% (v/v) formalin for 24 h.

The relative organs weights were calculated by Equation (2):

(2) $$\begin{array}{r} Organ\;relative\;weight\;\left(g\right)=\frac{Organ\;weight\;\left(g\right)}{Rat\;body\;weight\;\left(g\right)} \end{array}$$

### Biochemical Analyses

The complete blood cell count (RBCs, WBCs, Hb, Platelets, Hct, MCV, MCH, and MCHC) was determined. The iron forms represented [plasma iron, total iron-binding capacity (TIBC), and transferrin saturation (TS)] were determined. In addition, the plasma total protein, transferrin, ferritin, and albumin were determined. Furthermore, calcium and zinc were determined to evaluate their absorbance completion with iron. Glucose, plasma lipid profile, liver, and kidney function were assessed. All biochemical analyses were conducted according to Tietz Textbook and Tietz clinical Guide (44, 45).

### Histopathology Examination

Fixed samples from the liver, kidney, and spleen were washed and embedded in paraffin blocks and sectioned into 5–6 μm sections using Microtome (Reichert-Jung, Germany) for serial specimens then mounted on glass slides and stained with hematoxylin and eosin (H & E) stain (46). The slides were observed under a histological light microscope (Olympus, Tokyo, Japan) at 400X magnification.

### Statistical Analysis

The data were presented as mean values ± standard deviation. Statistical analysis was performed using one-way analysis of variance (ANOVA) followed by Duncan's test. The Physicochemical and microbiological properties were statistically analyzed by two-way analysis of variance (ANOVA) followed by a t-test (LSD). The differences were considered significant at (*p* ≤ 0.05) We used IBM SPSS Statistics 23 software program for statistical analyses [IBM Corp (47) IBM SPSS Statistics for Windows, Version 23.0. IBM Corp, Armonk, NY] with *p* ≤ 0.05 considered statistically significant.

## Results and Discussion

### Characteristics and Efficiency of Nanocapsules

Table 1. illustrates the encapsulation efficiency of the BSA nanoparticles (BSA-NPs). Results revealed that the sizes of the spherical nanoparticles of BSA-NPs, Fe@ BSA-NPs, FA@ BSA-NPs, and Fe + FA@ BSA-NPs were determined by dynamic light scattering were 52.85, 57.98, 77.59, and 206.40 nm, respectively, with a significant increase in the size when FA, Fe, or both FA and Fe were loaded. The polydispersity indices demonstrated their narrow size distribution, this confirmed the chemical cross-linking between tyrosine residues that could result in the overall stability of BSA-NPs and BSA-NPs loaded with Fe or FA (48).

**Table 1:** Characterization of the bovine serum albumin nanoparticles (BSA-NPs).

**Characteristics**	**BSA-NPs**	**Fe@ BSA-NPs**	**FA@ BSA-NPs**	**Fe + FA@ BSA-NPs**	**Free Fe**	**Free Fe + FA**
**Encapsulation efficiency of the bovine serum albumin nanoparticles (BSA-NPs)**						
Size (nm)	52.85 ± 1.7^d^	77.59 ± 2.2^b^	57.98 ± 1.1^c^	206.40 ± 83^a^	–	–
Calculated PDI	0.543^a^	0.421^b^	0.300^c^	0.291^d^	–	–
ζ potential (mv)	−22.10 ± 5^a^	−24.61 ± 1^b^	−30.30 ± 3^c^	−21.20 ± 3^a^	–	–
Encapsulation efficiency	–	95.78 ± 5.42^b^	97.54 ± 3.19^a^	95.78 & 97.54^*^	–	–
**Cytotoxicity assessment for applied forms (Hemolysis %)**						
5	–	1.18 ± 0.00^aA^	–	11.42 ± 0.01^aC^	3.54 ± 0.01^aB^	14.17 ± 0.02^aD^
10	–	2.36 ± 0.02^bA^	–	11.81 ± 0.00^bC^	6.69 ± 0.02^bB^	20.87 ± 0.01^bD^
15	–	10.63 ± 0.01^cB^	–	13.39 ± 0.03^cC^	8.27 ± 0.01^cA^	22.05 ± 0.01^cD^
50	–	12.20 ± 0.01^dB^	–	15.35 ± 0.01^dC^	9.84 ± 0.01^dA^	24.80 ± 0.00^dD^
100	–	15.35 ± 0.00^eA^	–	24.02 ± 0.01^eC^	22.44 ± 0.00^eB^	30.71 ± 0.01^eD^

*Data represented as means of triplicates ± SD. Means in the same column followed by different lower-case letters and means in the same row followed by different upper case letters are significantly different (p ≤ 0.05). PDI, Polydispersity index; BSA-NPs, Bovine Serum Albumin nanoparticles; FA, Folic Acid*.

* *As each of Fe and FA encapsulation efficiency were determined separately*.

Zeta potentiometry was used to estimate the surface charge of the NPs to determine their suspension stability. The results showed the zeta potential for BSA-NPs, Fe@ BSA-NPs, FA@ BSA-NPs, and Fe + FA@ BSA-NPs were −22.10, −30.30, −24.6, and −21.20 mV, suggesting strong repulsive forces and electrostatic stabilization between them. Electrostatically stabilized nanosuspensions have a maximum zeta potential of −30 mV, while the value for satirically stabilized nanosuspensions is ~ 20 mV (49). The higher encapsulation efficiency was for FA@ BSA-NPs and Fe@ BSA-NPs (97.54 and 95.78%, respectively). BSA can establish hydrophilic and hydrophobic bonds with numerous molecules (48).

### BSA-NPs Characterization

**Figure 1:**
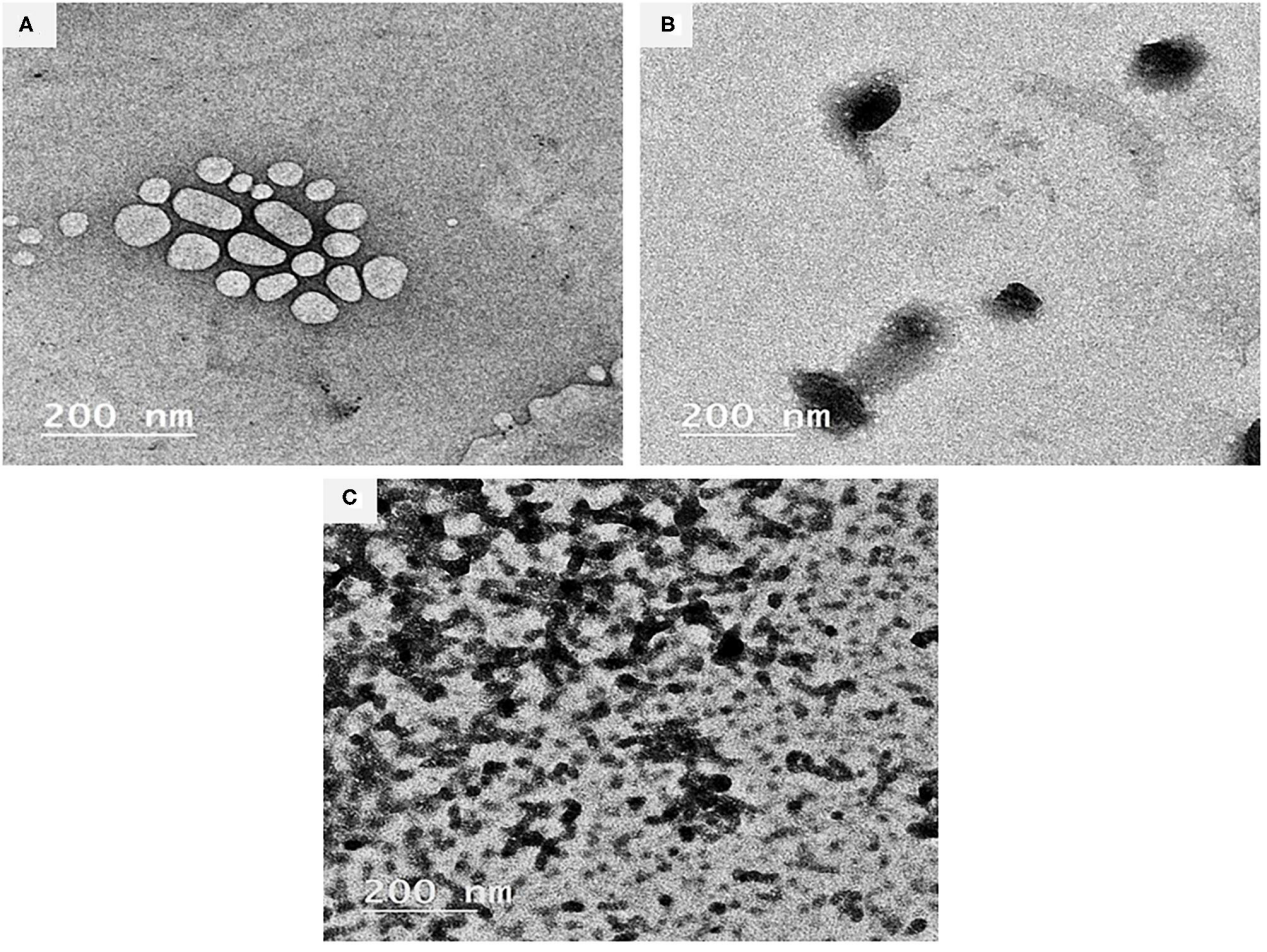
Characterization of BSA-NPs *via* Transmission Electron Microscope (TEM). **(A)** Bovine Serum Albumin nanoparticles (BSA-NPs). **(B)** Iron Bovine Serum Albumin nanoparticles (Fe@ BSA-NPs). **(C)** Folic Acid Bovine Serum Albumin nanoparticles (FA@ BSA-NPs).

Figures 1A–C illustrates the TEM images (200 nm, 100 kV, 20000X) of the BSA-NPs, Fe@ BSA-NPs, and FA@ BSA-NPs nanoparticles. TEM can provide a detailed microscopic structure consistent with the results obtained from the dynamic light scattering (DLS) of particle sizes. The bonding of the Fe and FA with BSA resulted in changes to the BSA structure as shown in the TEM images. Fe and FA nanoparticles (Figures 1B,C) were shown to possess spherical morphology with some aggregation indicating successful immobilization of BSA. Similar results were reported by Li et al. (50). In spite of what was previously reported—that nanoparticles disrupt the basic structure of BSA (51, 52)—Morozova et al. (53), when compared BSA with BSA-NPs, reported that the characteristics of the circular dichroism signal did not change in the BSA-NPs spectrum.

### Cytotoxicity Assessment of BSA-NPs by Hemolytic Activity Assay

Table 1 shows the cytotoxicity assessment (Hemolysis %) of applied forms (Fe and FA free and their nanocapsules). Hemolysis represents the most commonly employed initial toxicity assessment (54). The results revealed that that the percentage of hemolysis increased significantly by increasing concentrations and added FA to Fe either in free or nano-encapsulated forms. Furthermore, the comparison between the free and nano-encapsulated forms revealed the significant role of nanoencapsulation in reducing cytotoxicity. For instance, when comparing the highest examined concentration (100 μg/mL) of free Fe and Fe@ BSA-NPs, nanoencapsulation reduced hemolysis from 22.44 to 15.35%, respectively, and reduced the hemolysis of free Fe + FA from 30.71 to 24.02% in the Fe + FA@BSA-NPs from. This effect is well-documented, as nano-encapsulated drugs formulated BSA-NPs, enhanced cytotoxicity, and drug delivery (55, 56). The hemolytic activity results were used as a guide for safe fortification based on Amin and Dannenfelser (57), who stated that hemolysis values below 10% are considered non-hemolytic while values above 25% are considered to be hemolytic. Consequently, all forms of applied fortification concentrations in this study were kept below 50 μg mL^−1^ to ensure that the percent of hemolysis was within the safe range.

## SFY Physiochemical Characteristics

### Physiochemical Analyses

Fortification with Fe and FA, either free or nano-encapsulated, showed no effect on SFY's incubation time up to pH 4.6, as all batches reached pH 4.6 ± 0.1 after 6 h. This observation agrees with previously reported results (58). The changes in the pH, TA, and WHC values of SFY products during 21 days of cold storage are shown in Table 2. The results showed no significant differences between the treatments at the fresh product stage. The pH values showed a significant decrease (*p* ≤ 0.05) at 21 days of storage in all products, and values ranged from 4.58 to 4.37. The least pH value was recorded in T_2_ products fortified with free Fe at the 21st day of storage. The results are comparable to those reported by Santillan-Urquiza et al. (59).

**Table 2:** Physiochemical characteristics of SFY products during storage.

	**Storage period (days)**				
**Treatments**	**Fresh**	**7**	**14**	**21**	**Means**
**pH value**					
C	4.58 ± 0.01	4.55 ± 0.03	4.49 ± 0.01	4.44 ± 0.03	4.52^A^
T_1_	4.55 ± 0.04	4.52 ± 0.04	4.44 ± 0.01	4.39 ± 0.03	4.48^BC^
T_2_	4.53 ± 0.03	4.50 ± 0.03	4.42 ± 0.04	4.37 ± 0.01	4.46^C^
T_3_	4.58 ± 0.01	4.56 ± 0.04	4.48 ± 0.01	4.43 ± 0.03	4.51^A^
T_4_	4.57 ± 0.04	4.54 ± 0.03	4.47 ± 0.03	4.42 ± 0.03	4.50^AB^
Means	4.56^a^	4.53^b^	4.46^c^	4.41^d^	LSD = 0.027
**Titratable acidity (%)**					
C	0.86 ± 0.01	0.89 ± 0.01	0.91 ± 0.03	0.96 ± 0.03	0.91^B^
T_1_	0.88 ± 0.01	0.93 ± 0.03	0.96 ± 0.04	1.01 ± 0.04	0.95^A^
T_2_	0.90 ± 0.01	0.95 ± 0.03	0.97 ± 0.04	1.03 ± 0.04	0.96^A^
T_3_	0.86 ± 0.04	0.89 ± 0.01	0.92 ± 0.01	0.98 ± 0.03	0.91^B^
T_4_	0.86 ± 0.01	0.89 ± 0.01	0.92 ± 0.01	0.98 ± 0.03	0.91^B^
Means	0.87^c^	0.91^b^	0.94^b^	0.99^a^	LSD = 0.026
**Water holding capacity (%)**					
C	66.49 ± 0.38	64.80 ± 0.14	55.80 ± 0.14	47.20 ± 0.28	58.57^B^
T_1_	62.54 ± 0.42	58.74 ± 0.32	46.45 ± 0.21	41.74 ± 0.14	52.37^C^
T_2_	60.76 ± 0.28	53.34 ± 0.20	47.66 ± 0.28	42.34 ± 0.20	51.03^D^
T_3_	69.67 ± 0.42	66.22 ± 0.28	56.89 ± 0.28	47.34 ± 0.28	60.03^A^
T_4_	69.90 ± 0.35	66.92 ± 0.17	56.62 ± 0.17	46.92 ± 0.28	60.09^A^
Means	56.87^a^	62.00^b^	52.68^c^	45.11^d^	LSD = 0.258

*Data represented as means of duplicates ±SD. C, Control plain SFY; T_1_, SFY fortified with free Fe + ascorbic acid; T_2_, SFY fortified with free Fe + FA + ascorbic acid; T_3_, SFY fortified with Fe@BSA-NPs + ascorbic acid; T_4_, SFY fortified with Fe +FA @BSA-NPs + ascorbic acid. LSD, Least significant differences at 0.05 % level. Means with different superscripts in a raw or column are significantly different at p ≤ 0.05 level. BSA-NPs, Bovine Serum Albumin nanoparticles; FA, Folic Acid*.

The opposite trend was observed in the TA of all products that increased gradually during the storage period up to the 21st day. The TA of the SFY was significantly higher in T_1_ and T_2_ SFY products containing free Fe and Fe + FA compared to the control. Iron is an essential micronutrient in the metabolism of most living organisms, and increased viability, metabolic activity, and fermentation power in presence of Fe have been previously reported (60, 61). These results are consistent with those reported by Güven et al. (62).

SFY fortified with nanocapsules Fe@BSA-NPs and Fe + FA@BSA-NPs (T_3_ and T_4_) showed the highest WHC values, which were significantly higher than the control. On the other hand, yogurt products fortified with free Fe or Fe + FA (T_1_ and T_2_) showed a significant decrease (*p* ≤ 0.05) in WHC values on the 21st day of storage. These effects may be related to the pH decreasing below 4.6, contributing to casein rearrangement, water release (24), and the porous structure exhibited by the microstructure (Figures 5B,C). While functional yogurt fortified with nanocapsules showed more stability due to the NP size, this did not affect the yogurt microstructure (Figures 5D,E). These observations agreed with previously obtained results for Fe-fortified yogurt (9, 63, 64).

### Apparent Viscosity of SFY

**Figure 2:**
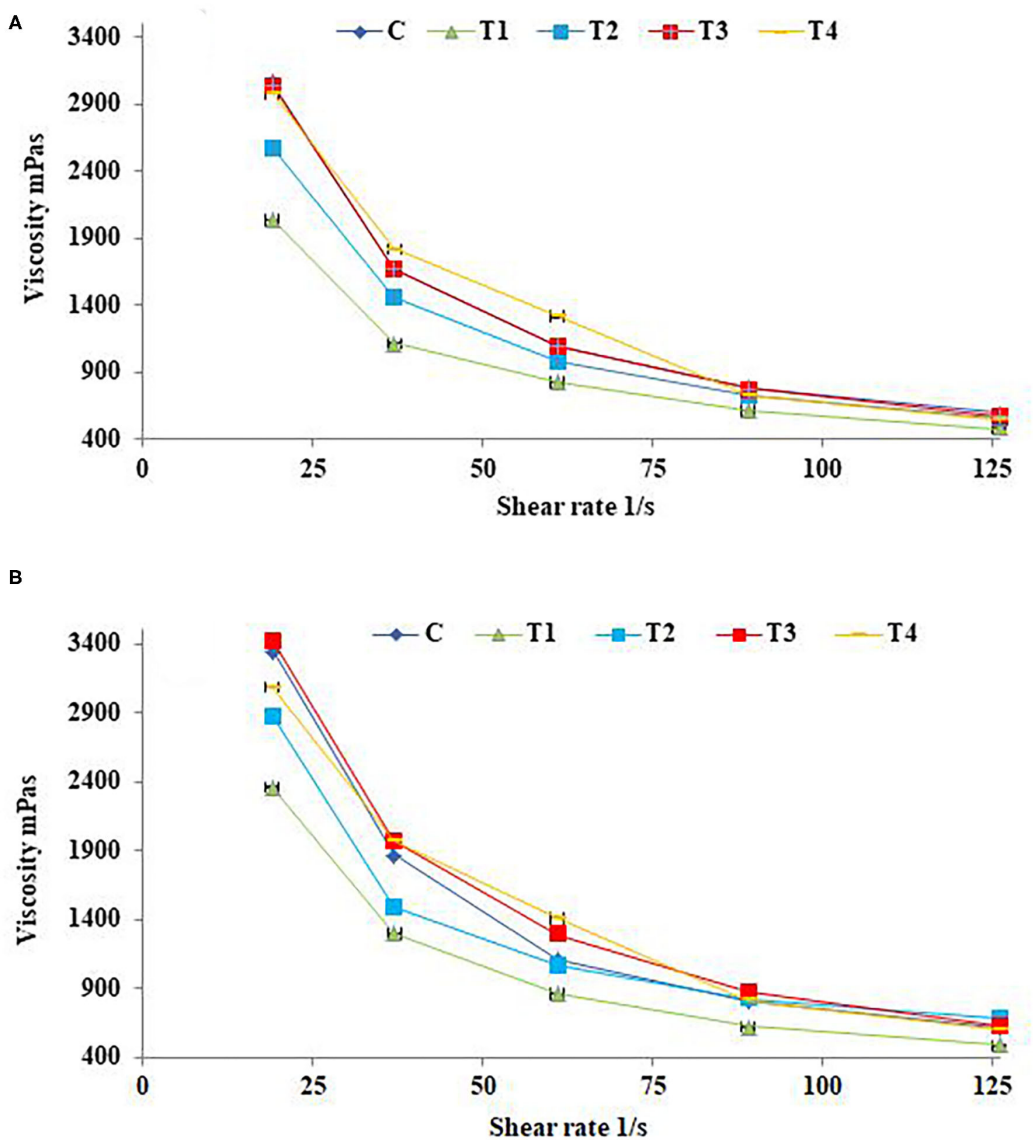
Apparent viscosity of stirred functional yogurt products at different shearing rates. **(A)** Fresh products. **(B)** After 21 days of cold storage. Data represented as means of duplicates ±SD. C, Control plain SFY; T_1_, SFY fortified with free Fe + ascorbic acid; T_2_, SFY fortified with free Fe + FA + ascorbic acid; T_3_, SFY fortified with Fe@BSA-NPs + ascorbic acid; T_4_, SFY fortified with Fe +FA @BSA-NPs + ascorbic acid. BSA-NPs, Bovine Serum Albumin nanoparticles; FA, Folic Acid.

The effects of shear rate on fresh SFY apparent viscosity after 21 days of storage are illustrated in Figures 2A,B. Yogurts exhibit a complex, shear-thinning, time-dependent flow behavior, and yogurt viscosity is therefore very important for processing, handling, process design, product development, and quality control aspects (65). The up-mode shear rates showed the SFY shear-thinning as the apparent viscosity of all samples was significantly reduced along with increasing the shear rate. There were differences between different treatments on the apparent SFY viscosity. Treatments T_3_ and T_4_ fortified with nanocapsules scored the highest values of apparent viscosity comparing to the control (C). While T_1_ and T_2_ fortified with free forms of Fe and Fe + FA, respectively, showed lower values. These results are consistent with the WHC values (Table 2), indicating that SFY products are a gel system of casein micelles with entrapped water (66). The apparent viscosity of all fortified SFY products showed slight increases in viscosity after 21 days of storage (Figure 2B). This may be attributed to the fact that the SFY viscosity was affected by the number, strength, structure, and spatial distribution of casein micelle bonds enhanced by storage (67).

### Color Analysis

The changes in the color parameters in fresh SFY treatments and after 21 days of storage are shown in Table 3. The products fortified with free Fe and Fe + FA (T_1_ and T_2_) were significantly darker, and the reddishness tended toward yellowishness (*p* ≤ 0.05) compared to the control. T_3_ and T_4_ showed decreased lightness (*L*) and increased *a* and *b* values. The color red-yellowish color differences were primarily due to the ferrous sulfate and the yellow color due to FA, which imparted some color when added in its free form. The organoleptic properties had a significant effect on the sensory evaluation results (Figure 6). On the other hand, products fortified with nanocapsules Fe@BSA-NPs and Fe + FA@BSA-NPs (T_3_ and T_4_) tended to be significantly lighter with higher *L* and lower *a* and *b* values (*p* ≤ 0.05) compared to T_1_ and T_2_. This may be due to the effect of nanoencapsulation on masking the color (10, 41). These results were confirmed by the sensory evaluation results (Figure 6). After 21 days of storage, there were significant decreases in *L* and *b* and an increased *a* value (*p* ≤ 0.05) in all products. During storage, the presence of Fe promoted the oxidation of lipids (Figure 4), consequently decreased lightness and changes in *a* and *b* values. These values are comparable to those reported by Gaucheron (6).

**Table 3:** Color parameters of SFY.

**Treatments**	**Storage period (days)**	L^*^	a^*^	b^*^
C	1	88.80^aB^ ± 0.28	−0.96^bA^ ± 0.04	8.67^aA^ ± 0.12
	21	88.49^bB^ ± 0.13	−0.75^aB^ ± 0.02	8.19^bA^ ± 0.05
T_1_	1	84.53^aA^ ± 0.09	0.57^bB^ ± 0.03	12.88^aC^ ± 0.14
	21	84.12^bA^ ± 0.01	1.98^aA^ ± 0.04	11.28^bC^ ± 0.07
T_2_	1	83.69^aA^ ± 0.20	0.46^bB^ ± 0.01	14.36^aC^ ± 0.16
	21	83.21^bA^ ± 0.04	1.53^aA^ ± 0.02	13.02^bC^ ± 0.02
T_3_	1	88.86^aB^ ± 0.26	−0.25^bC^ ± 0.02	9.79^aB^ ± 0.08
	21	88.78^bB^ ± 0.30	0.08^aD^ ± 0.01	9.27^bB^ ± 0.04
T_4_	1	88.82^aB^ ± 0.19	−0.32^bC^ ± 0.02	9.89^aB^ ± 0.08
	21	87.80^bB^ ± 0.14	0.02^aD^ ± 0.01	9.66^bB^ ± 0.07

*Data represented as means of duplicates ±SD. C, Control plain SFY; T_1_, SFY fortified with free Fe + ascorbic acid; T_2_, SFY fortified with free Fe + FA + ascorbic acid; T_3_, SFY fortified with Fe@ BSA-NPs + ascorbic acid; T_4_, SFY fortified with Fe +FA @ BSA-NPs + ascorbic acid*.

* *L, value represents lightness from black (0) to white (100), a, value represents color ranging from red (+) to green (–); b, value represents yellow (+) to blue (–). Means in the same column followed by different lower-case letters and means in the same row followed by different upper case letters are significantly different (p ≤ 0.05). BSA-NPs, Bovine Serum Albumin nanoparticles; FA, Folic Acid*.

### Phenolic, Flavonoid Content, and Antioxidant Potentials

**Figure 3:**
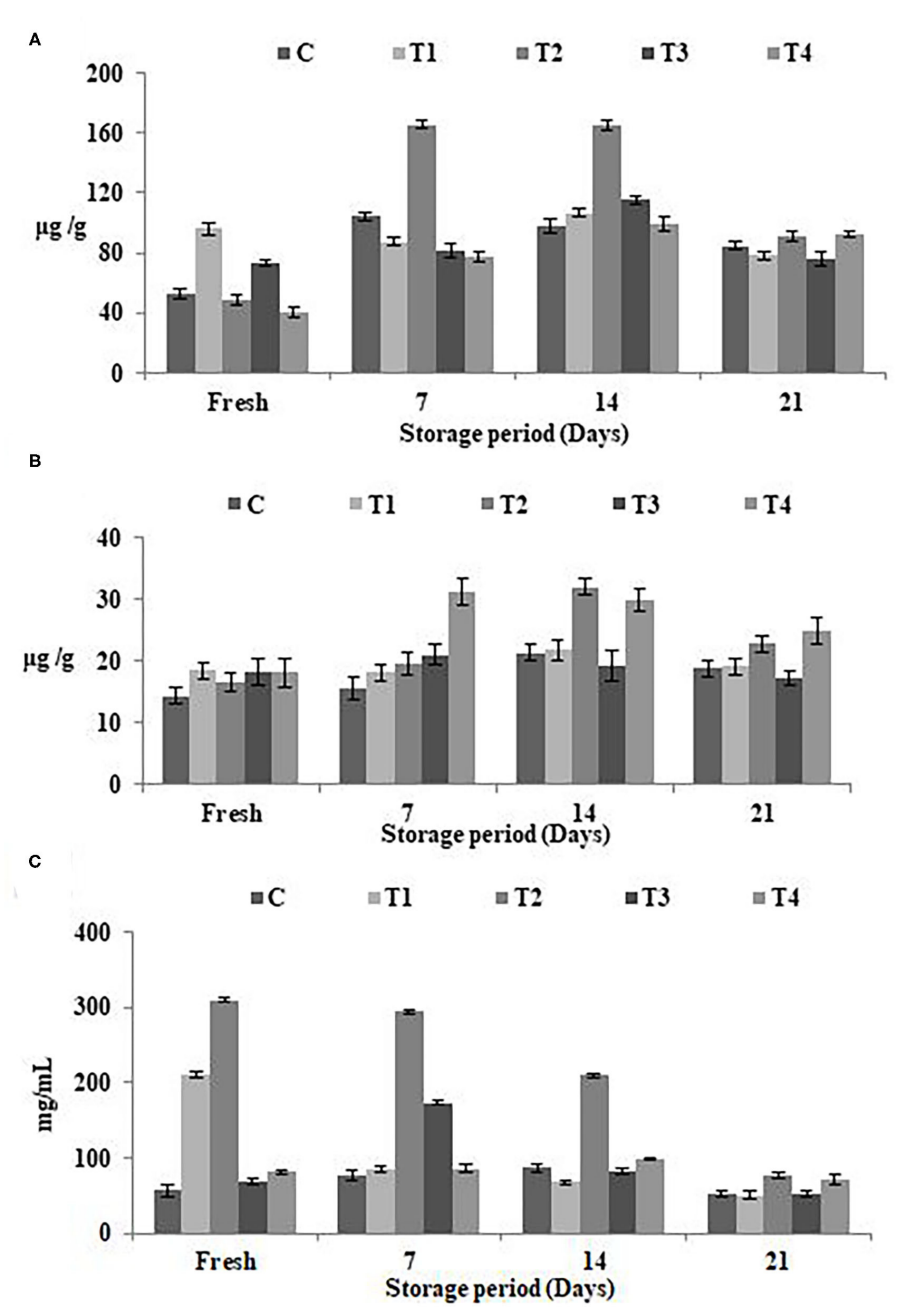
Phenolic, flavonoid content, and antioxidant potentials in SFY during storage. **(A)** Total phenolic content (TPC) (μg gallic acid equivalents/g sample). **(B)** Total flavonoid content (TFC) (μg catechol equivalents/g sample). **(C)** Antioxidant potentials represented as IC_50_ (mg mL^−1^), the inhibitory concentration at which 50% of DPPH radical is scavenged. Data represented are means of duplicates ±SD. C, Control plain SFY; T_1_, SFY fortified with free Fe + ascorbic acid; T_2_, SFY fortified with free Fe + FA + ascorbic acid; T_3_, SFY fortified with Fe@BSA-NPs + ascorbic acid; T_4_, SFY fortified with Fe +FA @BSA-NPs + ascorbic acid. BSA-NPs, Bovine Serum Albumin nanoparticles; FA, Folic Acid.

The estimated concentrations of total phenolic content (TPC), total flavonoid content (TFC), and antioxidant potentials in SFY with different fortifications were analyzed when fresh and during 21 days of cold storage Figures 3A–C. Figures 3A,B shows the increased TPC and TFC values with fortification, particularly in the free Fe + FA and Fe + FA@BSA-NP treatments (T_2_ and T_4_). The results showed the same trend in antioxidant potentials (Figure 3C). On the 21st day of the storage period a decline in TPC, TFC, and the SYF products' antioxidant effects was noticed. The results showed that the optimum shelf life of products is <14 days of cold storage. Similar patterns have been reported by Citta et al. (68).

### Lipid Oxidation

**Figure 4:**
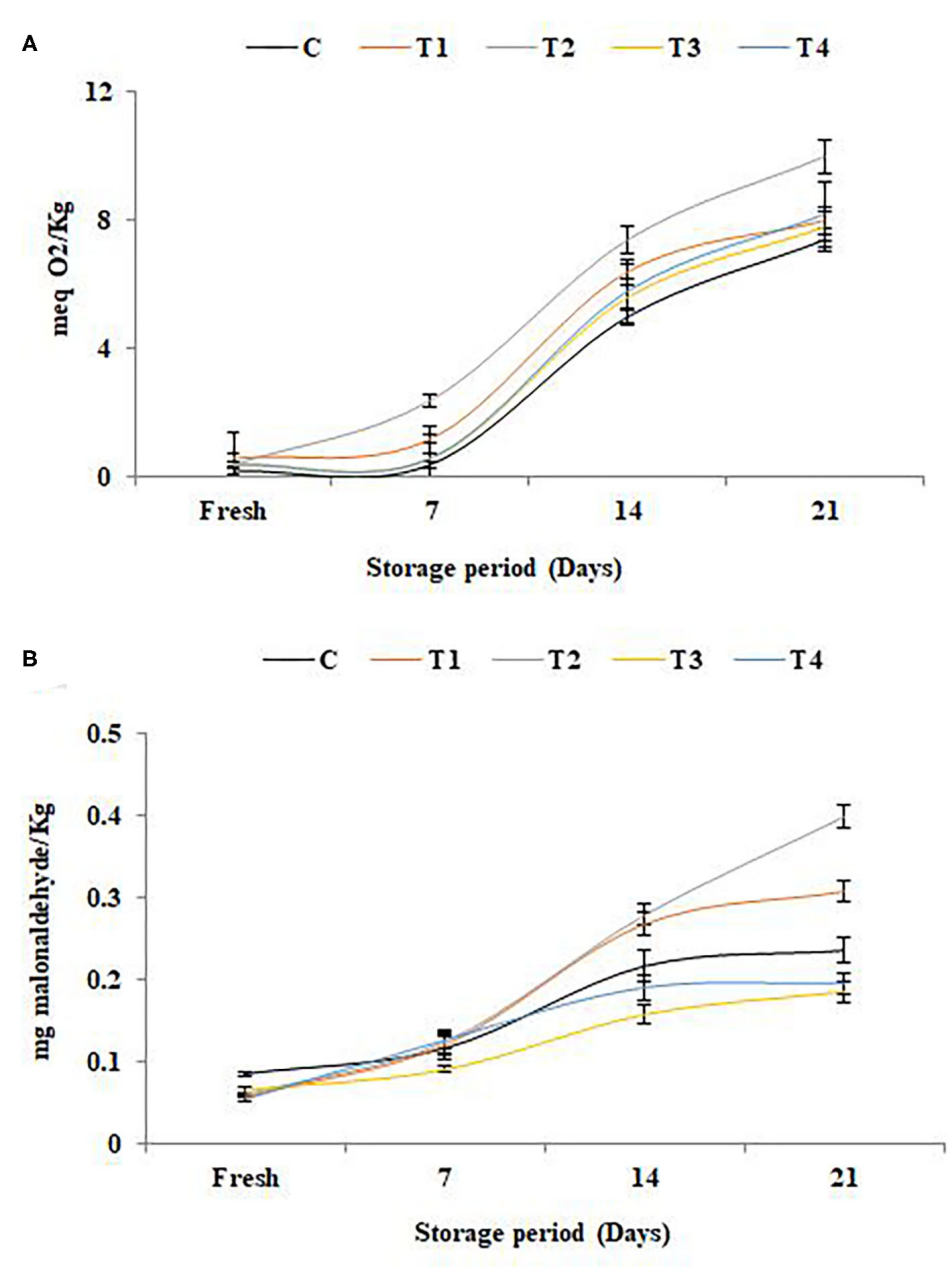
Lipid oxidation in stirred functional yogurt during storage. **(A)** Thiobarbituric acid reactive substances (TBARS) value. **(B)** Peroxide value. Data represented are means of duplicates ±SD. C, Control plain SFY; T_1_, SFY fortified with free Fe + ascorbic acid; T_2_, SFY fortified with free Fe + FA + ascorbic acid; T_3_, SFY fortified with Fe@BSA-NPs + ascorbic acid; T_4_, SFY fortified with Fe +FA @BSA-NPs + ascorbic acid. BSA-NPs, Bovine Serum Albumin nanoparticles; FA, Folic Acid.

The results of lipid oxidation in SFY during storage illustrated in Figures 4A,B were correlated to the antioxidant potential (Figure 3). The elevation in free radical peroxide oxygen (Figure 4A) caused an overproduction of malonaldehyde (MDA), a known marker of the final products of polyunsaturated fatty acids peroxidation (Figure 4B). The results indicated that the antioxidants in yogurt (Figure 3) are prone to protect the products against lipid peroxidation. These results are in agreement with Citta et al. (68). It was noteworthy that the products fortified with nanocapsules (T_3_ and T_4_) showed the least lipid oxidation due to the effect of nanoencapsulation on lipid protection. The protective role of nanoencapsulation has been previously documented (69).

### Microbiological Analysis of SFY

The changes in the viable counts of *S. thermophillus, L. bulgaricus, L. acidophilus*, and *B. bifidum* for the different yogurt products are shown in Table 4. In the fresh products, the *S. thermophillus, L. bulgaricus*, and *B. bifidum* counts for treatments were comparable to the control. These results are in agreement with those reported by Hekmat and McMahon (58). This microbial imbalance is a little more challenging compared to other dairy products because it may create an imbalance between the LAB and other bacteria Fe for their growth. The *L. acidophilus* count in SFY fortified with free supplements (T_1_ and T_2_) was higher than the control and nano-encapsulated supplements (T_3_ and T_4_) due to free Fe that activated microbial growth. *L. acidophilus* can uptake Fe to enhance its growth. These results were reflected in the TA, which showed higher values in T_1_ and T_2_ (Table 2), confirming the microbiological analysis results. The *S. thermophillus, L. bulgaricus, L. acidophilus*, and *B. bifidum* counts showed insignificant decreases during the storage period for all treatments. One of the most important factors affecting the viability of *S. thermophillus, L. bulgaricus, L. acidophilus*, and *B. bifidum* is acidity (35). However, up to the 21st day, the counts remained at levels sufficient for it to have beneficial effects as a probiotic by FAO/WHO standards (>6 log CFUg^−1^) (70). Moreover, no coliforms, yeasts, and molds were detected in any treatments either when fresh or throughout the storage period (data not shown), However, yeasts and molds started to be detected after 21 days of storage in the samples fortified with free supplements (T_1_ and T_2_).

**Table 4:** Microbiological analysis (Log_10_ CFUg^−1^) of SFY during storage.

	**Storage period (days)**				
**Treatment**	**Fresh**	**7**	**14**	**21**	**Means**
***S. thermophilus***					
C	7.42 ± 0. 10	7.39 ± 0.08	7.25 ± 0.07	7.05 ± 0.06	7.28^A^
T_1_	7.44 ± 0.11	7.41 ± 0.08	7.30 ± 0.06	7.10 ± 0.03	7.31^A^
T_2_	7.45 ± 0.08	7.42 ± 0.10	7.33 ± 0.07	7.12 ± 0.03	7.33^A^
T_3_	7.42 ± 0.11	7.39 ± 0.10	7.24 ± 0.06	7.07 ± 0.01	7.28^A^
T_4_	7.43 ± 0.11	7.40 ± 0.10	7.27 ± 0.06	7.09 ± 0.01	7.30^A^
Means	7.43^a^	7.40^a^	7.28^b^	7.09^c^	LSD = 0. 082
***L. bulgaricus***					
C	7.18 ± 0.10	7.16 ± 0.10	7.12 ± 0.07	6.88 ± 0.01	7.08^A^
T_1_	7.19 ± 0.10	7.18 ± 0.08	7.15 ± 0.07	6.93 ± 0.03	7.11^A^
T_2_	7.20 ± 0.11	7.18 ± 0.10	7.15 ± 0.08	6.95 ± 0.04	7.12^A^
T_3_	7.23 ± 0.13	7.20 ± 0.10	7.12 ± 0.07	6.83 ± 0.03	7.09^A^
T_4_	7.24 ± 013	7.22 ± 0.10	7.13 ± 0.07	6.82 ± 0.03	7.10^A^
Means	7.21^a^	7.19^a^	7.13^a^	6.88^b^	LSD = 0.088
***L. acidophilus***					
C	7.15 ± 0.10	7.13 ± 0.08	7.06 ± 0.06	6.86 ± 0.01	7.05^B^
T_1_	7.39 ± 0.13	7.38 ± 0.11	7.30 ± 0.08	7.11 ± 0.06	7.30^A^
T_2_	7.40 ± 0.13	7.39 ± 0.11	7.33 ± 0.07	7.15 ± 0.03	7.31^A^
T_3_	7.14 ± 0.08	7.11 ± 0.07	7.02 ± 0.04	6.88 ± 0.03	7.04^B^
T_4_	7.16 ± 0.08	7.13 ± 0.07	7.06 ± 0.06	6.92 ± 0.03	7.06^B^
Means	7.25^a^	7.23^a^	7.15^b^	6.98^c^	LSD = 0.083
***B. bifidum***					
C	7.15 ± 0.10	7.12 ± 0.08	6.94 ± 0.04	6.71 ± 0.01	6.98^A^
T_1_	7.14 ± 0.10	7.12 ± 0.08	6.92 ± 0.04	6.75 ± 0.01	6.98^A^
T_2_	7.16 ± 0.10	7.15 ± 0.10	6.96 ± 0.04	6.73 ± 0.02	7.00^A^
T_3_	7.15 ± 0.09	7.13 ± 0.08	6.96 ± 0.07	6.74 ± 0.07	6.99^A^
T_4_	7.17 ± 0.07	7.15 ± 0.07	6.98 ± 0.04	6.75 ± 0.02	7.01 ^A^
Means	7.15^a^	7.13^a^	6.95^b^	6.74^c^	LSD = 0.073

*Data represented as means of duplicates ±SD. C, Control plain SFY; T_1_, SFY fortified with free Fe + ascorbic acid; T_2_, SFY fortified with free Fe + FA + ascorbic acid; T_3_, SFY fortified with Fe@ BSA-NPs + ascorbic acid; T_4_, SFY fortified with Fe +FA @ BSA-NPs + ascorbic acid. LSD, Least significant differences at 0.05 % level. Means with different superscripts in a row or column are significantly different at p ≤ 0.05 level. BSA-NPs, Bovine Serum Albumin nanoparticles; FA, Folic Acid*.

### Microstructural Characterization of SFY

**Figure 5:**
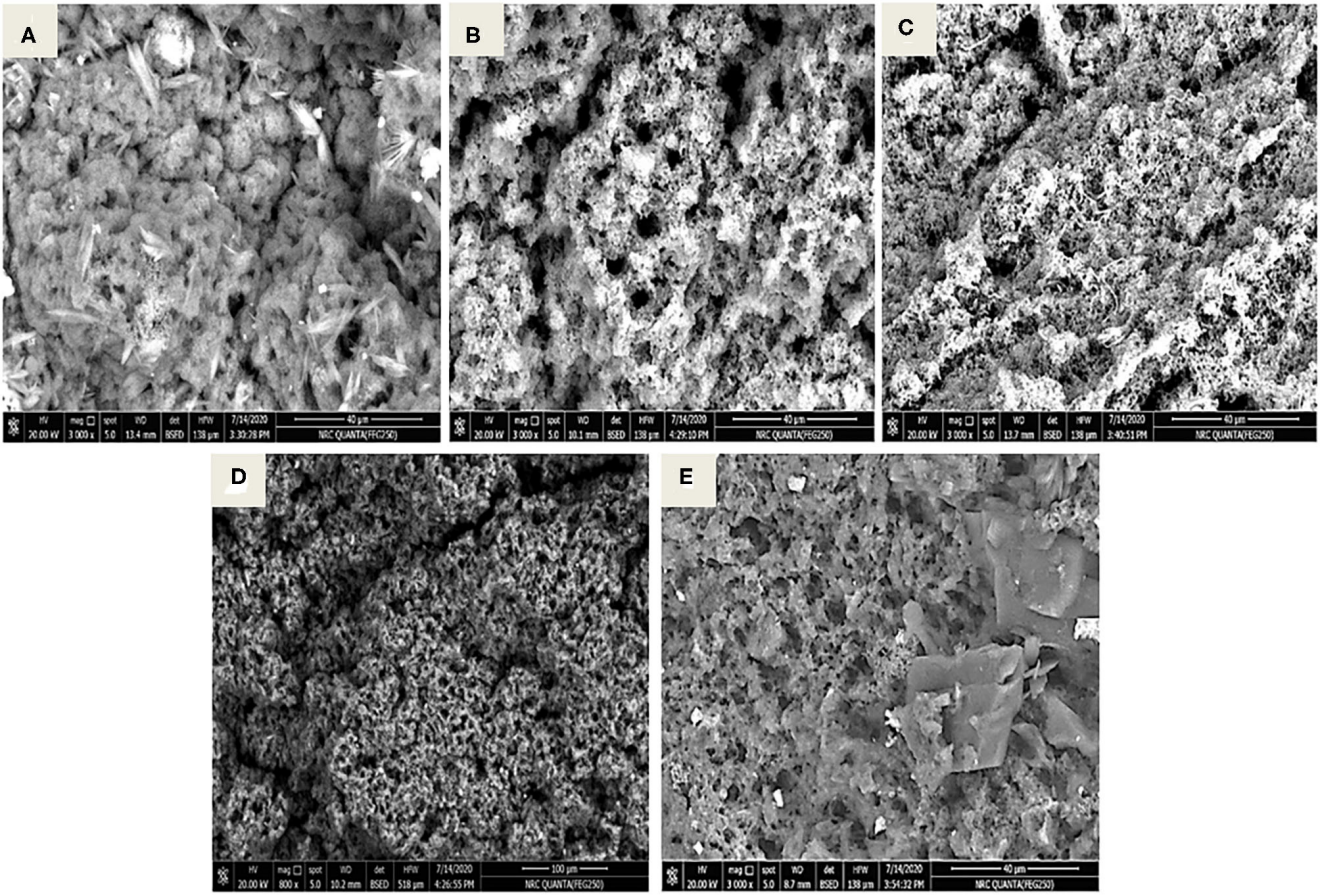
Micrographs of stirred functional yogurt products (X500, 50 μm, 10 kV). **(A)** Control plain SFY. **(B)** SFY fortified with free Fe + ascorbic acid (T_1_). **(C)** SFY fortified with free Fe + FA + ascorbic acid (T_2_). **(D)** SFY fortified with Fe@ BSA-NPs + ascorbic acid (T_3_). **(E)** SFY fortified with Fe +FA @ BSA-NPs + ascorbic acid (T_4_). BSA-NPs, Bovine Serum Albumin nanoparticles; FA, Folic Acid.

**Figure 6:**
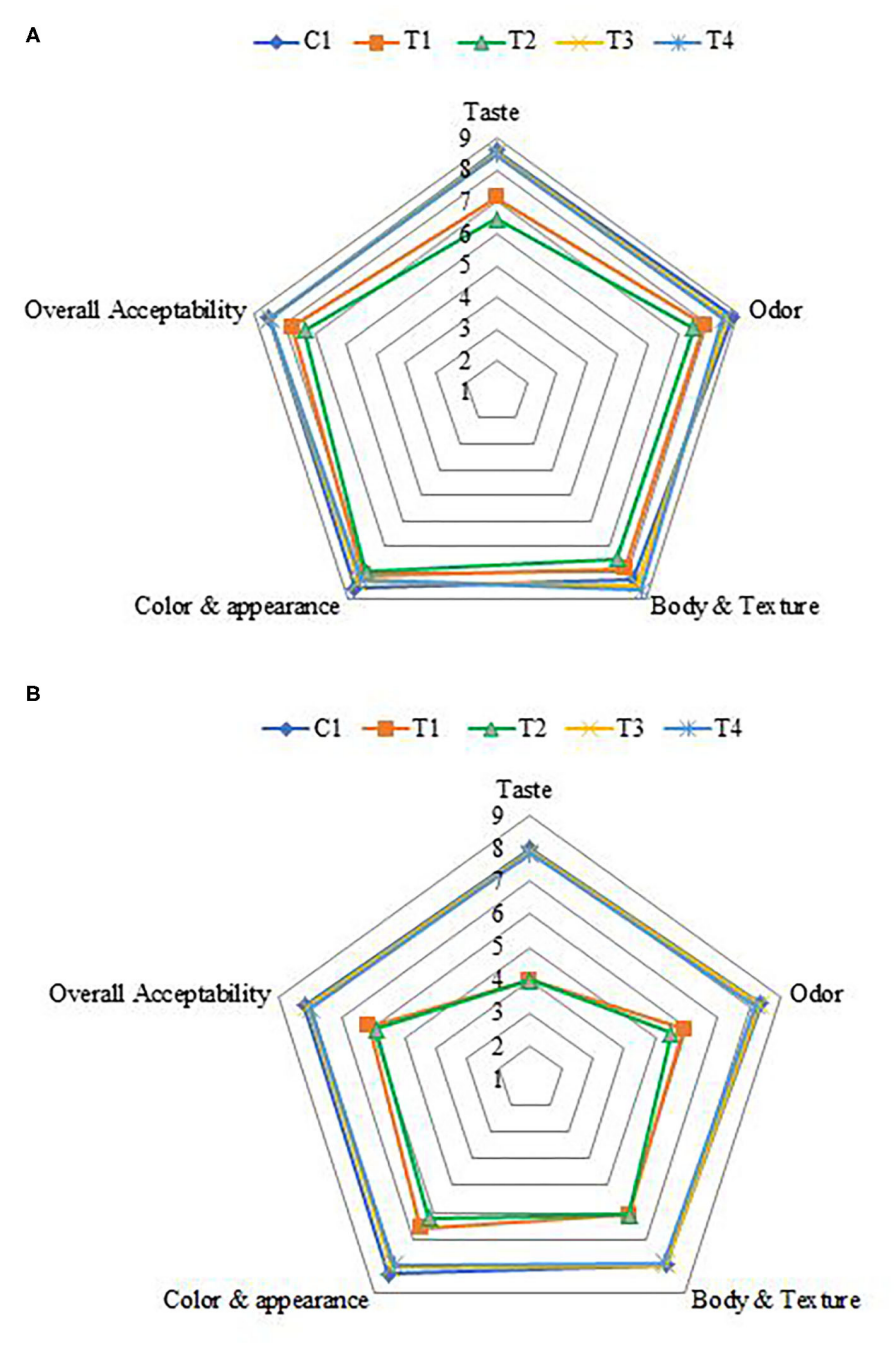
Sensory evaluation of stirred functional yogurt. **(A)** Fresh products. **(B)** After 21 days of cold storage. C, Control plain SFY; T_1_, SFY fortified with free Fe + ascorbic acid; T_2_, SFY fortified with free Fe + FA + ascorbic acid; T_3_, SFY fortified with Fe@-BSA-NPs + ascorbic acid; T_4_, SFY fortified with Fe +FA @-BSA-NPs + ascorbic acid. BSA-NPs, Bovine Serum Albumin nanoparticles; FA, Folic Acid.

Figures 5A–E shows the SEM micrographs of SFY products at 500X, 50 μm, and 10 kV. In the micrograph (Figure 5A), the control SFY appeared relatively uniform with a smooth surface. The micrographs in Figures 5B,C indicated that the microstructure of the free-supplement fortified products (T_1_ and T_2_) were affected, showing a clumpy porous structure that reflected the decreased WHC values (Table 2). On the other hand, nanocapsule-fortified products (T_3_ and T_4_) exhibited an intact microstructure with fewer gaps between the particles and were softer and with higher WHC (Table 2). These results were verified by the sensory evaluation (Figure 6) that showed that the nanocapsule-fortified products (T_3_ and T_4_) were comparable to the control. Similar results reported positive effects of nanoencapsulation on microstructure have been reported by El-Kholy (41).

### Sensory Evaluation

The effects of fortification on the sensory properties of SFY in the four fortified treatments comparing to plain control when fresh and after 21 days of storage are illustrated in Figures 6A,B. Overall, the acceptability scores of products fortified with nanocapsules (T_3_ and T_4_) showed great similarity to the plain control compared to products fortified with free supplements (T_1_ and T_2_). Obtained results agreed with previous researchers who reported that fortification with microencapsulated ferrous sulfate did not show notable effects on sensory properties and was similar to the control (71, 72). No changes were observed in the sensory parameters or acceptance after 21 days of cold storage in products fortified with nanocapsules (T_3_ and T_4_). These results indicated that the nano-encapsulation effectively reduced the negative changes in taste and odor, thus improving the sensory properties of Fe and Fe + FA. These results agreed with Kim et al. (12), who reported that Fe and ascorbic acid microcapsules are an effective means of fortification and can fortify dairy products without any changes in sensory parameters. In contrast, 21 days of cold storage negatively affected the taste and odor of SFY fortified with free supplements (T_1_ and T_2_), and this was especially due to the effect of lipids oxidation (Figure 4) on flavor. This observation agrees with Gaucheron (6), who reported that fortification with Fe complex causes oxidation-related off-flavor. It also negatively affected the products' color by imparting reddish-yellowness color to the SFY fortified with free supplements (T_1_ and T_2_), which was confirmed by color analyses (Table 3).

## Biological Experiment

### Impact of Fe Fortified Yogurt Diet on Body and Relative Organ Weights

The relative weight gain and organ weights of the liver, kidney, spleen, heart, and lungs of the rat groups are exhibited in Table 5. The results indicated that the relative body weights of the iron-deficiency anemia (IDA)-induced rats (G2) were severely affected and showed a significant decrease that reached 38%, while the groups (G3–G6) treated with fortified SFY showed varied significant increases in the relative body weights compared to induced group G2. G4 that received SFY fortified with free Fe + FA showed a significant weight increase of up to 35% with no mentioned effects on relative organ weights. It was noteworthy that G3 that received the free Fe supplement showed the highest relative organs weights, which included the liver, kidney, spleen, and lungs at 4.23, 0.84, 0.88, and 0.86 g, respectively, compared with negative control G1, positive control G2, and the other treated groups G4–G6. Ferrous sulfate is a permitted Fe supplement food additive in many countries; however, it causes undesirable side effects. It is necessary to develop new Fe supplements to increase Fe utilization and reduce its side effects (73). Nanotechnology is a means of decreasing the side effects while increasing its bioavailability (74).

**Table 5:** Fortified SFY impact on relative organs weights and plasma biochemical profile of treated rats.

**Parameters**	**Units**	**G1**	**G2**	**G3**	**G4**	**G5**	**G6**
**Relative weight gain and organs weights**							
Weight gain		13.51 ± 3.73^b^	8.81 ± 2.32^c^	14.42 ± 4.03^b^	19.58 ± 1.56^a^	15.79 ± 2.86^ab^	14.37 ± 2.07^b^
Liver		3.53 ± 027^a^	3.79 ± 0.55^a^	4.23 ± 1.39^a^	3.52 ± 0.32^a^	3.71 ± 0.48^a^	3.39 ± 0.37^a^
Kidney		0.63 ± 0.09^a^	0.72 ± 0.23^a^	0.84 ± 0.08^a^	0.74 ± 0.08^a^	0.76 ± 0.16^a^	0.74 ± 0.06^a^
Spleen		0.52 ± 0.16^a^	0.70 ± 0.20^a^	0.88 ± 0.44^a^	0.73 ± 0.11^a^	0.83 ± 0.43^a^	0.66 ± 0.14^a^
Heart		0.46 ± 0.01^a^	0.46 ± 0.14^a^	0.40 ± 0.04^a^	0.38 ± 0.01^a^	0.42 ± 0.03^a^	0.36 ± 0.02^a^
Lungs		0.77 ± 0.03^a^	0.84 ± 0.28^a^	0.86 ± 0.14^a^	0.78 ± 0.02^a^	0.84 ± 0.33^a^	0.82 ± 0.11^a^
**Plasma biochemical profile**							
**Glucose**	mg dL^−1^	203.25 ± 30.70^a^	169.00 ± 31.60^a^	187.75 ± 46.89^a^	185.00 ± 38.56^a^	192.50 ± 31.09^a^	169.75 ± 20.96^a^
**Plasma lipid profile**							
T.Ch.	mg dL^−1^	90.50 ± 7.89^bc^	94.00 ± 12.19^ab^	74.75 ± 7.93^c^	88.25 ± 6.18^bc^	89.75 ± 5.73^bc^	108.75 ± 16.60^a^
TG	mg dL^−1^	182.25 ± 10.62^a^	105.50 ± 9.88^b^	76.50 ± 13.07^b^	107.00 ± 16.43^b^	99.50 ± 13.10^b^	128.75 ± 75.07^b^
HDL	mg dL^−1^	32.00 ± 4.08^c^	45.00 ± 5.35^ab^	35.50 ± 7.85^bc^	42.25 ± 8.05^abc^	40.00 ± 3.36^bc^	51.00 ± 9.76^a^
LDL	mg/dL^−1^	22.75 ± 3.30^b^	29.25 ± 10.04^ab^	27.75 ± 7.63^ab^	28.25 ± 3.59^ab^	29.25 ± 4.03^ab^	33.75 ± 7.41^a^
**Liver function**							
AST	IU L^−1^	195.5 ± 051.00^a^	214.50 ± 48.23^a^	176.75 ± 15.10^a^	189.75 ± 31.60^a^	170.50 ± 17.21^a^	173.25 ± 26.98^a^
ALT	IU L^−1^	27.75 ± 5.12^b^	41.75 ± 11.08^a^	30.75 ± 3.20^b^	42.00 ± 6.21^a^	28.503.10^b^	29.50 ± 2.08^b^
ALP	IU L^−1^	288.75 ± 23.94^a^	287.50 ± 24.44^a^	241.50 ± 27.04^a^	262.25 ± 34.09^a^	293.5 ± 42.16^a^	264.25 ± 41.32^a^
T. Bilirubin	g dL^−1^	0.2175 ± 0.10^a^	0.2325 ± 0.02^a^	0.2075 ± 0.03^a^	0.2275 ± 0.02^a^	0.2850 ± 0.11^a^	0.2350 ± 0.07^a^
**Kidney function**							
Urea	mg dL^−1^	27.75 ± 3.59^b^	37.00 ± 4.96^a^	34.00 ± 3.36^a^	26.50 ± 5.06^b^	27.00 ± 1.82^b^	27.75 ± 1.70^b^
Creatinine	μmol L^−1^	0.985 ± 0.08^a^	0.970 ± 0.10^a^	0.960 ± 0.07^a^	0.983 ± 0.16^a^	0.975 ± 0.17^a^	0.983 ± 0.10^a^

*Data represented are means of relative weights (g) (n = 5) ±SD. Means in the same row followed by different letters are significantly different (p ≤ 0.05). A, G1 Negative control; B, G2 Positive control Group; C, G3 Fed SFY fortified with free Fe + ascorbic acid; D, G4 Fed SFY fortified with free Fe + FA + ascorbic acid; E, G5 Fed SFY fortified with Fe@ BSA-NPs + ascorbic acid; F, G6 Fed SFY fortified with Fe + FA@ BSA-NPs + ascorbic acid. BSA-NPs, Bovine Serum Albumin nanoparticles; FA, Folic Acid; T.Ch, Total cholesterol; TG, Triglycerides; HDL, High-density lipoprotein; LDL, Low-density lipoprotein; AST, Aspartate aminotransferase; ALT, Alanine aminotransferase; ALP, Alkaline phosphatase*.

### Monitoring Fortification Effects on Complete Blood Count

**Figure 7:**
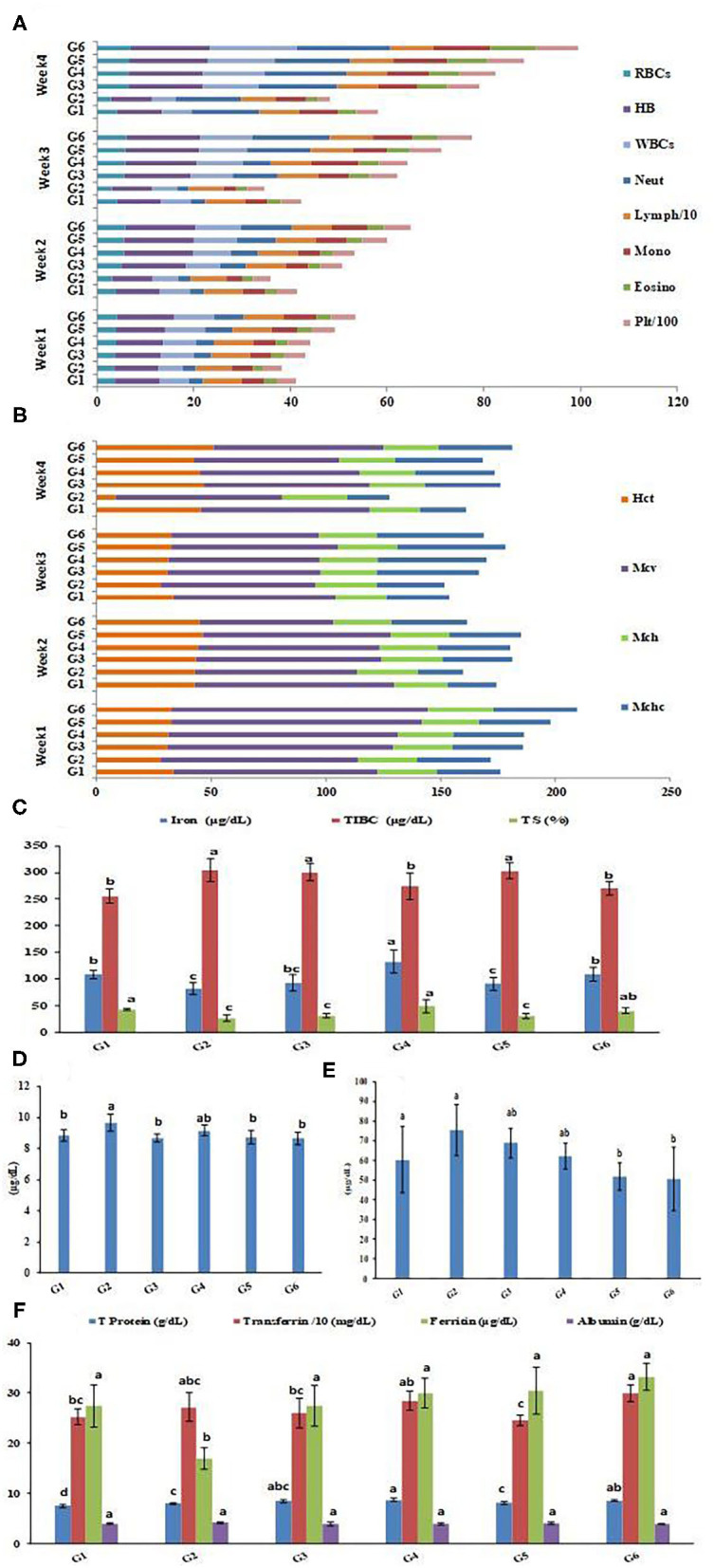
CBC (4 weeks of treatment) and serum profile of rats groups. **(A)** Complete blood cell count (CBC). **(B)** Red cell indices. **(C)** Iron parameters in rats groups. **(D)** Calcium concentrations in rats groups. **(E)** Zinc concentrations in rats groups. **(F)** protein parameters in rats groups. Serum profile data represented are means of duplicates ±SD. Serum profile data with different letters are significantly different (*p* ≤ 0.05). G1, Negative control; G2, Positive control Group; G3, Fed SFY fortified with free Fe + ascorbic acid; G4, Fed SFY fortified with free Fe + FA + ascorbic acid; G5, Fed SFY fortified with Fe@ BSA-NPs + ascorbic acid; G6, Fed SFY fortified with Fe + FA@ BSA-NPs + ascorbic acid. RBCs (10^6^ μL^−1^), WBCs (10^3^ μL^−1^), Hb (g dL^−1^), Platelets (μm^3^); TIBC, Total iron-binding capacity; TS, Transferrin saturation; BSA-NPs, Bovine Serum Albumin nanoparticles; FA, Folic Acid.

Complete blood cell count (CBC) and red cell indices of rat groups throughout the 4 weeks of treatment are exhibited in Figures 7A,B. Figure 7A illustrates the complete blood count represented in red blood cells (RBCs), protein hemoglobin (Hb), white blood cells (comprising neutrophils, lymphocytes, monocytes, and eosinophils), and platelets of the rat groups throughout the 4 weeks of treatment. The data revealed that the IDA was reflected in all blood parameters and showed decreased levels in the anemia-induced rat group G2 compared to the healthy control group G1. Treatments with Fe supplemented SFY products (G3–G6) achieved gradual enhancements in all blood parameters throughout the 4 weeks of feeding. In the fourth week of treatment, the RBCs counts in the treated groups G3, G4, G5, and G6 were 11.40, 12.70, 13.80, and 18 10^6^ μL^−1^, respectively, compared to the 6.17 10^6^ μL^−1^ in the healthy control group G1. The same pattern was recorded for red cell indices illustrated in Figure 7B. This regeneration of blood cells was correlated with the significantly elevated Fe (93, 132.5, 91, and 109.25 μgdL^−1^), compared to the induced group G2 (81.75 μgdL^−1^) and ferritin (27.5, 30, 30.5, and 33.25 μgdL^−1^) levels, the induced group G2 (17.025 μgdL^−1^) and transferrin levels (259.75, 284.5, 246, and 299.75 mg dL^−1^), and the control (252.75 mg dL^−1^), as illustrated in Figures 7C,F, which showed the same pattern as the blood cell count. The restoration of blood Hb from (8.30 gdL^−1^) in the IDA induced group G2 was more pronounced in G5 and G6, which received Fe nanocapsules (16.27 and 16.53 gdL^−1^, respectively), and this was more than in groups G3 and G4, which received the free forms with recorded Hb levels (15.48 and 15.53 gdL^−1^, respectively). These results encourage recommending nano-encapsulation as an efficient form of Fe delivery, especially in IDA treatment. Targeted drug delivery refers to nanocarriers carrying drugs to organs, tissues, and cells through local or systemic blood circulation, which allows the drugs to act directly on the targeted disease sites and thus more effectively generating their curative effects (75). No adverse effects on blood parameters or indices were recorded, which is unlike the significant changes that were reported for WBC, HCT, MCV, and MCHC in hematology parameters after NP treatment (76). This may refer to size-, dose-, and coating-dependent uptake of NPs (77). Additionally, the overall stability shown by the physicochemical characterization of BSA-NPs loaded with Fe or FA (Table 1) may interlink with its safe consumption results.

### Fortification Effects on Serum Profile of Rat Groups

Figures 7C–F exhibits the impact of fortification on the serum mineral and protein profiles of the rat groups. Figure 7C indicated that serum Fe parameters: Fe and transferrin saturation (TS) were negatively affected, showing the significant decreases reached (81.75 μgdL^−1^ and 26.67%, respectively) in the IDA-induced group (G2). Treatments with different Fe forms in SFY products succeeded in restoring serum Fe levels with varied responses. The highest serum Fe content was recorded for G4 (132.5 μgdL^−1^), which was significantly higher than the healthy control rat group G1 (108.5 μgdL^−1^), followed by G6 (109.25 μgdL^−1^), which was comparable to control group G1. The serum calcium and zinc concentrations were inversely correlated with the Fe concentrations, as shown in Figures 7D,E, respectively. The Fe-Ca and Fe-Zn interactions and competition in absorbance have been previously documented; over a range of “physiological” calcium intakes, Fe absorption was inversely correlated to the calcium content of the meal (78). Additionally, the Fe:Zn molar ratio was shown to be an important determinant of Fe-Zn interactions. A significant reduction in Zn absorption from Zn solution in water was observed when Fe:Zn was 25:1 (79).

The serum protein profile Figure 7F shows different patterns. Ferritin is a blood cell protein-containing Fe that stores Fe in tissues and is a well-known IDA marker (2). The rat groups' ferritin concentrations showed the lowest content (17.03 μgdL^−1^) in the IDA group (G2); its content was enhanced significantly in varying degrees in rats treated with different forms of Fe in SFY products (G3–G6) compared to the control. The free ferrous sulfate form (G3) (27.5 μgdL^−1^) succeeded in restoring ferritin to the normal healthy control group level (G1) (27.43 μgdL^−1^), while the free Fe + FA and nano-encapsulated forms, Fe@BSA-NPs, and Fe + FA@BSA-NPs exceeded the normal levels (30, 30.5, and 33.25 μgdL^−1^, respectively). A similar pattern was shown for total protein and transferrin. However, the albumin levels were not significantly affected. These results support the earlier reported hypothesis that nanoencapsulation is a feasible approach for targeted site-specific delivery of materials and efficient absorption through cells in the digestive system that improve bioavailability and solubility (69).

### Histopathology Examination of the Liver, Kidney, and Spleen

**Figure 8:**
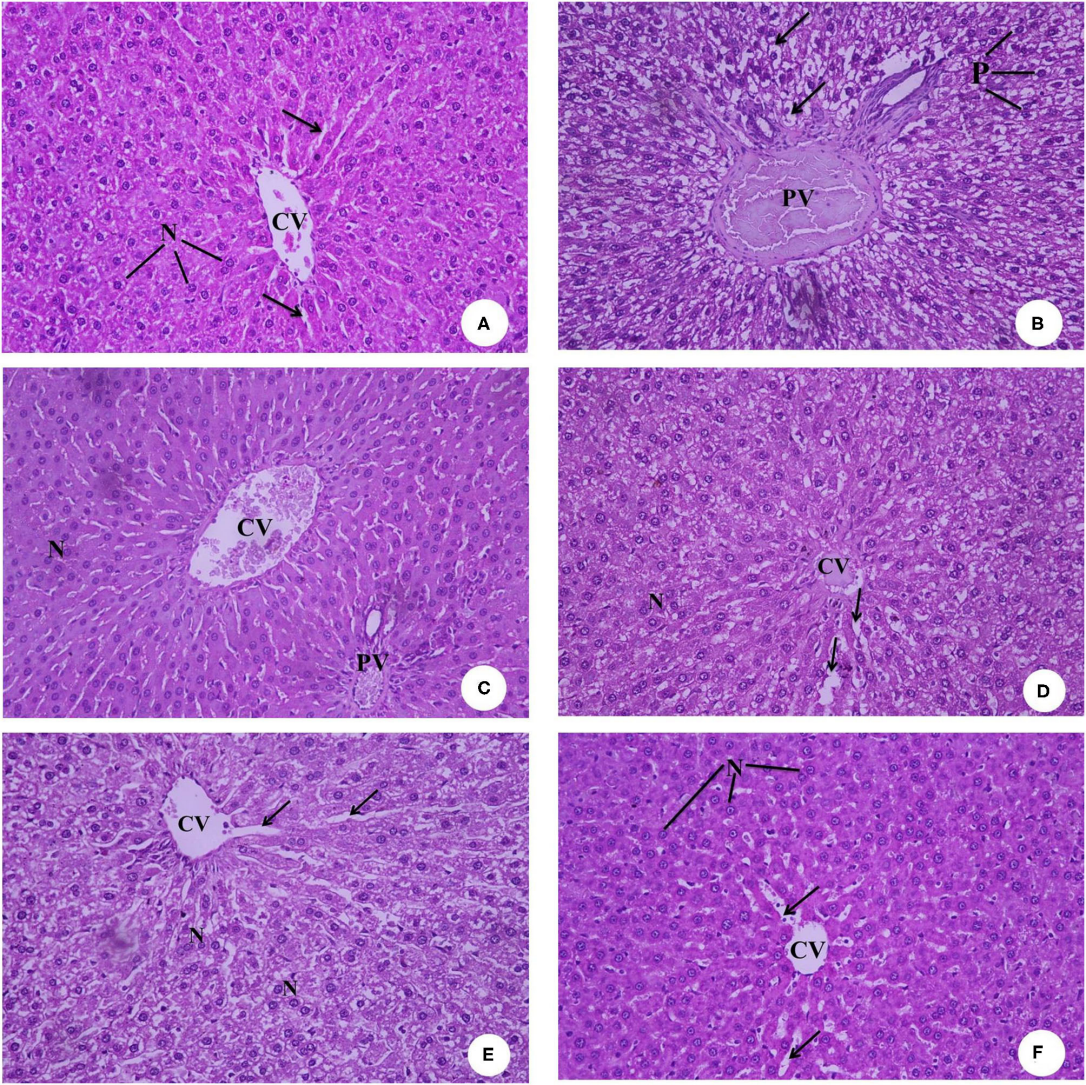
**(A–F)** Representative photomicrographs of liver sections of rats from the different experimental groups (Hematoxylin and eosin stain X 40). **(A)** G1 Negative control. **(B)** G2 Positive control group. **(C)** G3 Fed SFY fortified with free Fe + ascorbic acid. **(D)** G4 Fed FSY fortified with free Fe + FA + ascorbic acid. **(E)** G5 Fed SFY fortified with Fe@ BSA-NPs + ascorbic acid. **(F)** G6 Fed SFY fortified with Fe + FA@ BSA-NPs + ascorbic acid. BSA-NPs, Bovine Serum Albumin nanoparticles; FA, Folic Acid.

Figures 8A–F presents the hematoxylin and eosin-stained histopathology of the rat livers in the experimental groups. The photomicrograph of the negative control rats group (G1) in Figure 8A shows the normal histological architecture, central vein (CV), hepatocytes with round basophilic nuclei (N), and eosinophilic cytoplasm and blood sinusoids (arrows). Figure 8B of the IDA rat group (G2) shows a dilated and congested portal vein (PV), degenerated hepatocytes with pyknotic nuclei (P), and vacuolated cytoplasm (arrows). Figure 8C, which is the photomicrograph of the anemic rat group treated with SFY fortified with free Fe + ascorbic acid (G3), shows a relatively dilated and congested central vein (CV), congested portal vein (PV), and normal hepatocytes with round basophilic nuclei (N). The Figure 8D liver section of the rat group treated with SFY fortified with free Fe + FA +ascorbic acid (G4) shows a relatively improved liver architecture and central vein (CV) compared with the induced group G2, normal hepatocytes nuclei (N), and blood sinusoids (arrows). Figures 8E,F of rat groups treated with SFY fortified with nanocapsules (G5 and G6), respectively, shows restored and improved liver architecture, normal central vein (CV), hepatocytes with round basophilic nuclei (N) eosinophilic cytoplasm, and normal blood sinusoids (arrows). Histological examination of the livers confirmed the biochemical results in which the IDA rat group (G2) showed higher levels of aspartate aminotransferase (AST) (214.50 IUL^−1^) and analine aminotransferase (ALT) (41.75 IUL^−1^) values compared to the control (195.50 and 27.75 IUL^−1^, respectively) (Table 5).

**Figure 9:**
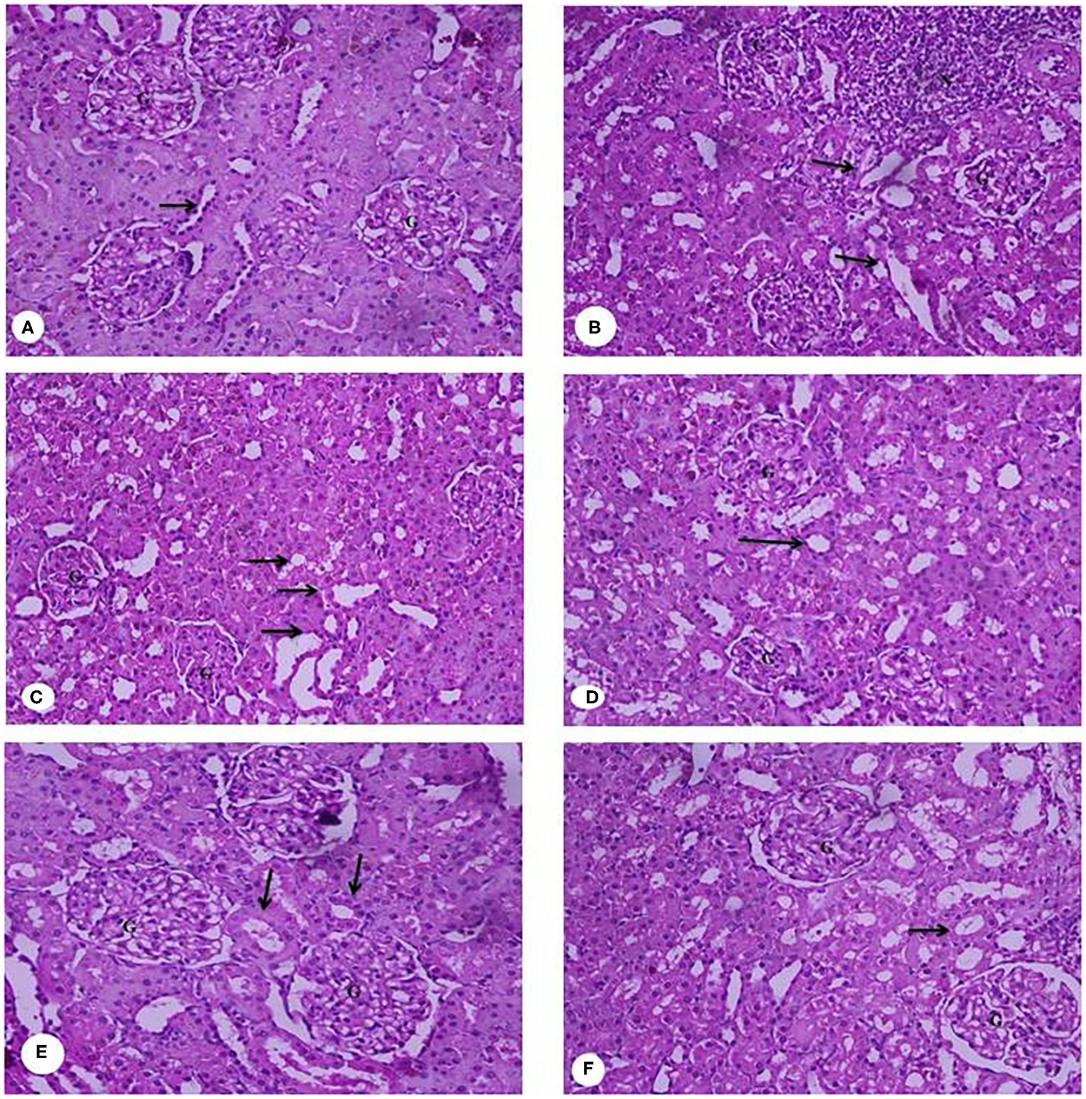
**(A–F)** Representative photomicrographs of kidney sections of rats from the different experimental groups (Hematoxylin and eosin stain X 40). **(A)** G1 Negative control. **(B)** G2 Positive control group. **(C)** G3 Fed SFY fortified with free Fe + ascorbic acid. **(D)** G4 Fed SFY fortified with free Fe + FA + ascorbic acid. **(E)** G5 Fed SFY fortified with Fe@ BSA-NPs + ascorbic acid. **(F)** G6 Fed SFY fortified with Fe + FA@ BSA-NPs + ascorbic acid. BSA-NPs, Bovine Serum Albumin nanoparticles; FA, Folic Acid.

Figures 9A–F presents the rats' kidney photomicrographs from different groups. Figure 9A shows the negative control group (G1) kidney section with a normal renal cortex and glomerulus (G) and normal proximal and distal convoluted tubules (arrow). Figure 9B is of IDA-induced rats (G2), showing infiltration of inflammatory cells surrounding the distorted glomeruli and tubules (arrow). The kidney sections of rats treated with yogurt fortified with free Fe (G3) (Figure 9C) show tubular dilatation associated with some vacuoles around the renal tubules (arrow). For Figure 9D, the kidney sections of rats treated with free Fe+ FA (G4) show kidney architecture, glomerulus (G) with a normal capsule, and the proximal and distal tubules and the collecting ducts (arrows). Figures 9E,F kidney sections of rats treated with nanocapsules (G5) and (G6), respectively, shows restored and improved kidney architecture and renal glomeruli with normal structure. The tubules have a relatively regular, distinct lumen. The lobular organization of the glomerulus and a flat epithelium lining the glomerular capsule can be seen (arrows). The histopathological kidney examination was consonant with the kidney function analysis indicating elevated urea levels (37 mg dL^−1^) in the IDA group (G2), while in groups G5 and G6 treated with encapsulated supplements (22 and 27.75 mg dL^−1^, respectively), the urea levels were comparable to the control (27.75 mg dL^−1^) (Table 5). Ranjan et al. (80) reported infiltration in liver tissue and tubular degeneration and tubular dilation in kidney cells for ingested nanoparticles. These effects are dose dependent and relative to accumulation and toxicity to local as well as distant organs (77).

**Figure 10:**
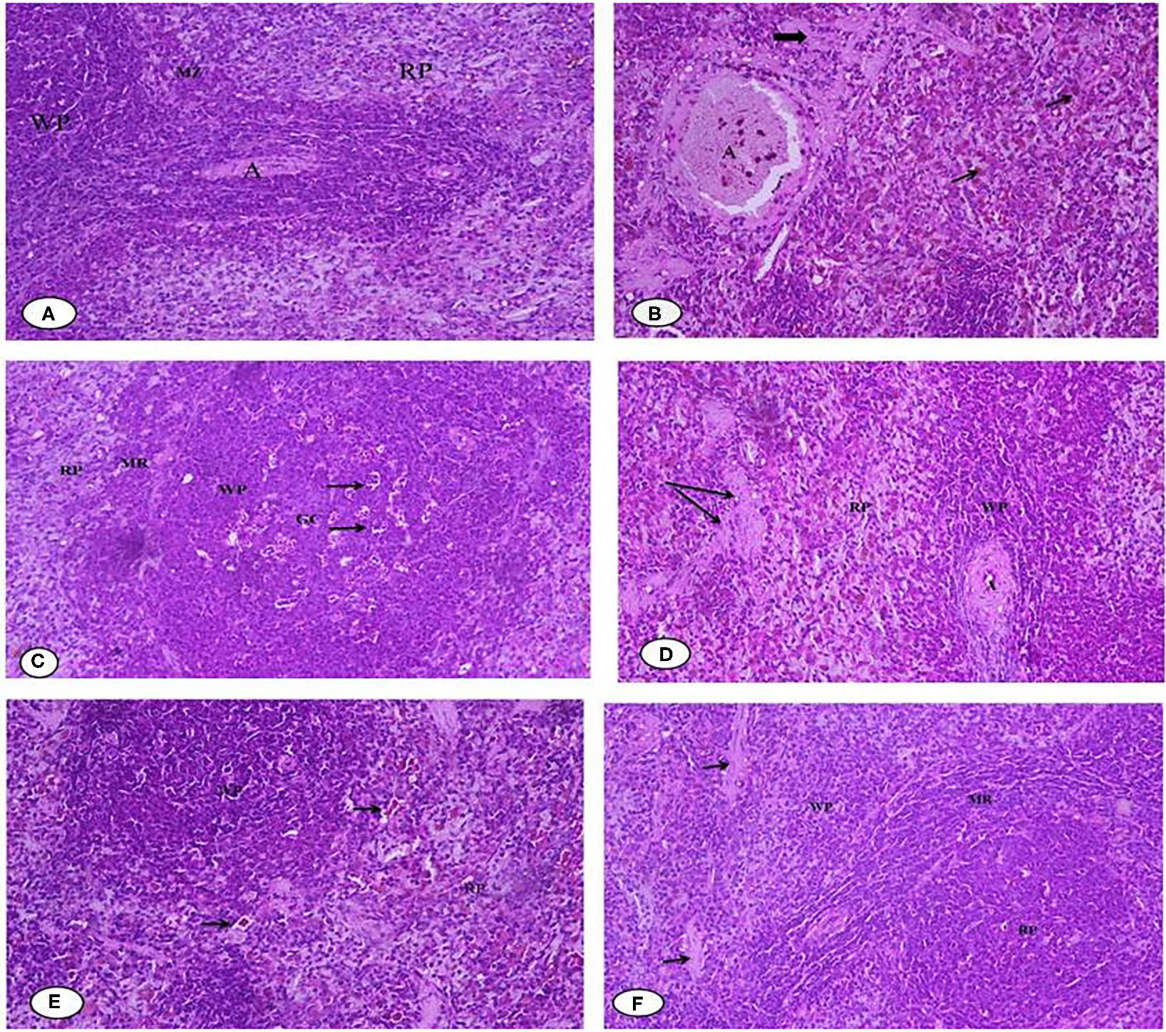
**(A–F)** Representative photomicrographs of spleen sections of rats from the different experimental groups (Hematoxylin and eosin stain X 40). **(A)** G1 Negative control. **(B)** G2 Positive control Group. **(C)** G3 Fed SFY fortified with free Fe + ascorbic acid. **(D)** G4 Fed SFY fortified with free Fe + FA + ascorbic acid. **(E)** G5 Fed SFY fortified with Fe@ BSA-NPs + ascorbic acid. **(F)** G6 Fed SFY fortified with Fe + FA@ BSA-NPs + ascorbic acid. BSA-NPs, Bovine Serum Albumin nanoparticles; FA, Folic Acid.

Figures 10A–F illustrates the histopathology of the rats' spleen in the different groups. Figure 10A of the negative control (G1) spleen section shows the normal architecture of the splenic pulps, which are the white pulps (WP) formed from closely packed lymphocytes and containing a central arteriole (A) and the red (RP) pulps formed from the splenic cords separated by the blood sinusoids marginal zone. Figure 10B of the positive control (G2) shows that most of the white pulp (WP) cells exhibit degenerative changes in the form of vacuolated cytoplasm and numerous sub-capsular clear spaces (thick arrow) and few others containing hemolyzed RBCs (thin arrow) between the splenic parenchyma. The vacuolization occurred in the wall of the central artery (A). For Figure 10C, the section of rat spleens treated with free Fe (G3) shows the active germinal center of the white pulp with a predominance of the cell nest appearance of the macrophages (arrow) and increased number of large macrophages in the marginal zone (MR). Figure 10D shows spleen sections of rats treated with free Fe+ FA (G4), showing the white pulps (WP), marginal zone (MR), red pulps (RP), and slightly dilated sinusoids (arrow) with the relatively normal architecture of the central artery (A). Figure 10E spleen sections of rats treated with encapsulated Fe (G5) show improved red pulps and white pulp (WP) with its central arteriole (CA), and considerable blood extravasation (arrow) deposition was noticed. For Figure 10F, the spleen section of rats treated Fe and FA nanocapsules (G6) shows that the splenic tissues appeared almost normal and manifested a tendency toward recovery. Some significant signs toward complete vasculature and tissues recovery were observed in the red pulp (RP), white pulp (WP), and marginal zone (MR) compared with the previously examined groups in which no areas of hemorrhage or hemolysis were observed; however, a few space areas between the sinusoids were observed (arrow). However, the rat groups' splenetic changes did not affect the blood glucose levels as all groups showed blood glucose levels lower than negative control G1 (Table 5). Products of the spleen (insulin antibodies, inhibitors, and norepinephrine) were reported to play an acute role in carbohydrate metabolism and the formation of glycogen in the liver from lactic acid (81, 82).

In conclusion, we performed histological examinations of the liver, kidney, and spleen and found no significant organ or tissue architectural alterations in the rat groups (G5 and G6) that ingested nano-encapsulated supplements. These results agree with Karabasz et al. (83), who reported the safety and efficiency of nutrient delivery *via* organic nanocapsules. However, it is important to obtain detailed information and verify the safety of nanomaterials before use in human clinical trials (84).

## Conclusions

To sum up, the chemical cross-linking confirmed the overall stability of BSA-NPs and BSA-NPs loaded with Fe or FA with successful immobilization of BSA as shown in TEM images. The freeform of iron- and folic acid-fortified SFY products surpassed the nano-encapsulated form in restoring most of the monitored plasma iron parameters with less competition with zinc absorbance. But, first and foremost, they negatively affected the liver, kidney, and spleen, which were announced in biochemical parameters, and had additional effects on lipid oxidation, microstructure, viscosity, and sensory properties. Nanocapsule-fortified SFY restored the blood count, iron, and protein parameters with no adverse effects or architectural alterations in the liver, kidney, or spleen as biochemical or histological examinations indicated. Furthermore, they showed enhanced viscosity, water-holding capacity, microstructure, the least lipid oxidation, and overall sensorial acceptability. Based on obtained results, bovine serum albumin-nanoparticles (BSA-NPs) of iron (Fe) and folic acid (FA) can be recommended as anti-anemia supplement in different functional food applications.

## Data Availability Statement

The raw data supporting the conclusions of this article will be made available by the authors, without undue reservation.

## Ethics Statement

The sensory evaluation of the nano-encapsulated iron and folic acid fortified functional yogurt products studied in the manuscript was conducted at Food Technology Department, Arid Lands Cultivation Research Institute (ALCRI), City of Scientific Research and Technological Applications (SRTACity), under the supervision and agreement of the Institutional Committee. The ethics committee waived the requirement of written informed consent for participation. The animal study was reviewed and approved by Alexandria University Ethical Committee (AlEXU-IACUC), a member of International Council for Laboratory Animal Science (ICLAS) (Permission number: AU08200415362).

## Author Contributions

AD, TS, HE, and WE-K were responsible for the conceptualization, experimental design, methodology and performed the formal analyses, and data curation and analysis. AD, TS, and WE-K wrote the original draft of the manuscript. All authors contributed equally to this research work, revised, edited, and approved the manuscript.

## Conflict of Interest

The authors declare that the research was conducted in the absence of any commercial or financial relationships that could be construed as a potential conflict of interest.
